# Supplementary material for: Dynamics and Determinants of SARS-CoV-2 RT-PCR Testing on Symptomatic Individuals Attending Healthcare Centers during 2020 in Bahia, Brazil
Source: Viruses. 2022 Jul 15;14(7):1549. doi: 10.3390/v14071549 (PMC9321627; doi:10.3390/v14071549)
Supplement: Supplementary file 1 [file viruses-14-01549-s001.zip › File S1-PCR Bahia BR V4 VIRUSES ISSUE - models.pdf]

Dynamics and determinants of SARS-CoV-2 RT-PCR testing on symptomatic individuals attending healthcare centers during 2020 in Bahia, Brazil.

Supplementary Text S1 - Model variants, odds ratios, code example

1) Model variants

1.1) Model 1: RT-PCR positive rate (Figure 4)

This model estimated the positive rate per week. It used dataset (ii) (see **RT-PCR sub datasets** for details) starting at week 15, when test numbers started to rise.

Variables considered:

Variable	Meaning	Type
time_group	Week of 2020	categorical
municipio_IDEB	IDEB index of municipality of individual	numeric
municipio_density	Population density of municipality of individual	numeric
municipio_percap_PIB	GDP of municipality of individual	numeric
age	Age of individual	numeric
sample_type	Type of sample	categorical
kit_test	Test kit used	categorical
gender	Gender of individual	categorical
race	Race of individual	categorical
laboratory	Laboratory performing the test	categorical
SARSCOV2	Test result	numeric

Model terms considered:

GAM term	Optional
s(time_group,bs='re') + s(age) + s(sample_type,bs='re') + s(kit_test,bs='re')+ s(gender,bs='re') + s(race,bs='re') + s(municipio_IDEB) + s(municipio_density) + s(municipio_percap_PIB)	no *1
s(time_group,bs='re') + s(age) + s(time_group,age,bs='re')	yes*2
s(time_group,bs='re') + s(race,bs='re') + s(time_group,race,bs='re')	yes
s(time_group,bs='re') + s(gender,bs='re') + s(time_group,gender,bs='re')	yes
s(time_group,bs='re') + s(kit_test,bs='re') + s(time_group,kit_test,bs='re')	yes
s(time_group,bs='re') + s(municipio_IDEB) + s(time_group,municipio_IDEB,bs='re')	yes
s(time_group,bs='re') + s(municipio_density) + s(time_group,municipio_density,bs='re')	yes
s(time_group,bs='re') + s(municipio_percap_PIB)+ s(time_group,municipio_percap_PIB,bs='re')	yes
s(laboratory,bs='re')	yes
s(laboratory,kit_test,bs='re')	yes

A total of 512 model variants are possible with the terms listed above. Note that some terms in the above table could be redundant, for example ”s(time_group,bs='re') + s(age)” in *1 and *2. In these

cases, if *[2] would be included, the "s(time_group,bs='re') + s(age)" from *[1] would not, to avoid term repetitions. Models (model_structure) using a dataset Y were executed using the instruction:

bam(as.formula(model_structure), data = Y, family = 'binomial', nthreads=12, discrete = TRUE, control = gam.control(trace = F), select=TRUE)

The selected model variant was the first in the table below, where the two other best scoring models are presented:

**Model structure**	**AIC difference to selected model**
SARSCOV2 ~ s(age)+ s(gender,bs='re')+ s(kit_test,bs='re')+ s(laboratory,kit_test,bs='re')+ s(municipio_density)+ s(municipio_IDEB)+ s(municipio_percap_PIB)+ s(race,bs='re')+ s(sample_type,bs='re')+ s(time_group,bs='re')+ s(time_group,kit_test,bs='re')+ s(time_group,municipio_density,bs='re')+ s(time_group,municipio_IDEB,bs='re')+ s(time_group,municipio_percap_PIB,bs='re')+ s(time_group,race,bs='re')	0
SARSCOV2 ~ s(age)+ s(gender,bs='re')+ s(kit_test,bs='re')+ s(laboratory,bs='re')+ s(laboratory,kit_test,bs='re') + s(municipio_density)+s(municipio_IDEB)+ s(municipio_percap_PIB)+ s(race,bs='re')+ s(sample_type,bs='re')+ s(time_group,bs='re')+ s(time_group,kit_test,bs='re')+ s(time_group,m unicipio_density,bs='re')+ s(time_group,municipio_IDEB,bs='re')+ s(time_group,municipio_percap_PIB,bs='re')+ s(time_group,race,bs='re')	-0.001436740
SARSCOV2 ~ s(age)+ s(gender,bs='re')+ s(kit_test,bs='re')+ s(laboratory,bs='re')+ s(laboratory,kit_test,bs='re')+ s(municipio_density)+s(municipio_IDEB)+ s(municipio_percap_PIB)+ s(race,bs='re')+ s(sample_type,bs='re')+ (time_group,bs='re')+ s(time_group,gender,bs='re')+ s(time_group,kit_test,bs='re')+ s(time_group,municipio_density,bs='re')+ s(time_group,municipio_IDEB,bs='re')+ s(time_group,municipio_percap_PIB,bs='re')+ s(time_group,race,bs='re')	-0.006699656

The summary of the selected model:

```
Approximate significance of smooth terms:
                                   edf Ref.df    Chi.sq  p-value    
s(age)                           7.188      9 2.148e+03  < 2e-16 ***
s(gender)                        0.993      1 1.849e+02  < 2e-16 ***
s(kit_test)                      3.298      7 8.416e+05 0.010884 *  
s(kit_test,laboratory)          32.377     53 2.518e+05 0.102795    
s(municipio_density)             8.611      9 3.016e+03 1.21e-05 ***
s(municipio_IDEB)                8.759      9 3.385e+03  < 2e-16 ***
s(municipio_percap_PIB)          8.746      9 3.681e+03  < 2e-16 ***
s(race)                          2.207      4 3.869e+01 0.009446 ** 
s(sample_type)                   2.622      5 9.288e+02 6.44e-06 ***
s(time_group)                   26.081     33 3.422e+06 4.15e-06 ***
s(kit_test,time_group)          78.925    179 4.385e+05 0.000185 ***
s(municipio_density,time_group) 30.402     34 4.300e+04  < 2e-16 ***
s(municipio_IDEB,time_group)    28.698     34 2.863e+06  < 2e-16 ***
s(municipio_percap_PIB,time_group) 23.556  34 4.542e+04 0.001932 ** 
s(race,time_group)              40.081    166 3.516e+03 0.025183 *  
---
Signif. codes:  0 ‘***’ 0.001 ‘**’ 0.01 ‘*’ 0.05 ‘.’ 0.1 ‘ ’ 1
```

1.2) Model 2: RT-PCR test delay (Figure 5)

This model estimated the test delay (days) between sample collection and test result per week. It used dataset (ii) (see **RT-PCR sub datasets** for details) starting at week 15, when test numbers started to rise.

Variables considered:

Variable	Meaning	Type
time_group	Week of 2020	categorical
meso_patient	Meso-region of individual	categorical
age	Age of individual	numeric
sample_type	Type of sample	categorical
laboratory	Laboratory performing the test	categorical
delay_result	Time (days) between sample collection and test result	numeric

Model terms considered:

GAM term	Optional
s(time_group,bs='re') s(laboratory,bs='re') s(time_group,laboratory,bs='re')	no
s(meso_patient,bs='re')	yes
s(sample_type,bs='re')	yes
s(age)	yes *1
s(time_group,bs='re') + s(age) + s(time_group,age,bs='re')	yes *2
s(time_group,bs='re') + s(sample_type,bs='re') + s(time_group,sample_type,bs='re')	yes
s(time_group,bs='re') + s(meso_patient,bs='re') + s(time_group,meso_patient,bs='re')	yes

A total of 54 model variants are possible with the terms listed above. Note that some terms in the above table could be redundant, for example those marked with *1 and *2. In these cases, if *2 would be included, *1 would not, to avoid term repetitions. Models (model_structure) using a dataset Y were executed using the instruction:

bam(as.formula(model_structure), data=Y, family='poisson', nthreads = 12, discrete = TRUE, control = gam.control(trace = F), select=TRUE)

The selected model variant was the first in the table below, where the two other best scoring models are presented:

Model structure	AIC difference to selected model
delay_result ~ s(age)+ s(laboratory,bs='re')+ s(meso_patient,bs='re')+ s(meso_patient,laboratory,bs='re')+ s(sample_type,bs='re')+ s(time_group,bs='re')+ s(time_group,laboratory,bs='re')+ s(time_group,meso_patient,bs='re')+ s(time_group,sample_type,bs='re')	0

delay_result ~ s(age)+ s(laboratory,bs='re')+ s(meso_patient,bs='re')+ s(meso_patient,laboratory,bs='re')+ s(sample_type,bs='re')+ s(time_group,age,bs='re')+ s(time_group,bs='re')+ s(time_group,laboratory,bs='re')+ s(time_group,meso_patient,bs='re')+ s(time_group,sample_type,bs='re')	-0.08803636
delay_result ~ s(laboratory,bs='re')+ s(meso_patient,bs='re')+ s(meso_patient,laboratory,bs='re')+ s(sample_type,bs='re')+s(time_group,bs='re')+ s(time_group,laboratory,bs='re')+ s(time_group,meso_patient,bs='re')+ s(time_group,sample_type,bs='re')	-36.39048058

The summary of the selected model:

```
Approximate significance of smooth terms:
                                  edf Ref.df    Chi.sq p-value    
s(age)                      2.857e+00      9 1.337e+03 < 2e-16 ***
s(laboratory)               7.703e+00      8 6.776e+05 0.00188 ** 
s(meso_patient)             1.034e-03      6 3.200e-02 0.57629    
s(meso_patient,laboratory)  1.826e+01     48 3.456e+05 0.00122 ** 
s(sample_type)              1.239e+00      5 5.765e+03 0.04090 *  
s(time_group)               2.238e+01     33 5.612e+06 0.13178    
s(laboratory,time_group)    1.687e+02    242 7.398e+06 < 2e-16 ***
s(meso_patient,time_group)  1.490e+02    235 2.344e+05 < 2e-16 ***
s(sample_type,time_group)   2.230e+01    160 2.467e+04 0.08620 .  
---
Signif. codes:  0 '***' 0.001 '**' 0.01 '*' 0.05 '.' 0.1 ' ' 1
```

1.3) Model 3.i: Probability of positive RT-PCR dependent on time since symptoms (Figure 6A)

This model estimated the probability of a positive test dependent on time (days) since reported symptoms. It used dataset (iii) (see **RT-PCR sub datasets** for details in the main text), selecting only individuals who had at least one positive test and with a single reported date of symptoms. We also subset this dataset by considering only the time since symptoms for which more than 10 tests had been performed across the cohort.

Variables considered:

Variable	Meaning	Type
time_pso	Time (days) between collection date and reported symptoms date	numeric
age	Age of individual	numeric
sample_type	Type of sample	categorical
kit_test	Test kit used	categorical
laboratory	Laboratory performing the test	categorical
SARSCOV2	Test result	numeric

Model terms considered:

GAM term	Optional
s(time_pso,m=2)	no
s(time_pso,age,m=2,bs='fs')	yes
s(time_pso,sample_type,m=2,bs='fs')	yes
s(time_pso,kit_test,m=2,bs='fs')	yes
s(time_pso,laboratory,m=2,bs='fs')	yes

A total of 16 model variants are possible with the terms listed above. Models (model_structure) using a dataset Y were executed using the instruction:

bam(as.formula(model_structure), data=Y, family=binomial(link = 'logit'), nthreads=12, discrete = TRUE, control = gam.control(trace = F), select=TRUE)

The selected model variant was the first in the table below, where the two other best scoring models are presented:

Model structure	AIC difference to selected model
SARSCOV2 ~ s(time_pso,kit_test,m=2,bs='fs')+ s(time_pso,laboratory,m=2,bs='fs')+ s(time_pso,m=2)	0
SARSCOV2 ~ s(time_pso,kit_test,m=2,bs='fs')+ s(time_pso,laboratory,m=2,bs='fs')+ s(time_pso,m=2)+ s(time_pso,sample_type,m=2,bs='fs')	-1.153632e-04
SARSCOV2 ~ s(time_pso,age,m=2,bs='fs')+ s(time_pso,kit_test,m=2,bs='fs')+ s(time_pso,laboratory,m=2,bs='fs')+ s(time_pso,m=2)	-1.784376e+00

The summary of the selected model:

```
Approximate significance of smooth terms:
                         edf Ref.df Chi.sq p-value
s(time_pso,kit_test)   9.918     74 306.54  <2e-16 ***
s(time_pso,laboratory) 9.029     79  99.43  <2e-16 ***
s(time_pso)            3.800      9  42.80  <2e-16 ***
---
Signif. codes:  0 ‘***’ 0.001 ‘**’ 0.01 ‘*’ 0.05 ‘.’ 0.1 ‘ ’ 1
```

1.4) Model 3.ii: Probability of positive RT-PCR dependent on time since symptoms (Figure 6B)

This model estimated the probability of a positive test dependent on time (days) since reported symptoms. It used dataset (iii) (see **RT-PCR sub datasets** for details in the main text), selecting only individuals who had at least one positive test and with a single reported date of symptoms, further restricted to those for which the last test was negative. We also subset this dataset by considering only the time since symptoms for which more than 10 tests had been performed across the cohort.

Variables considered:

Variable	Meaning	Type
time_pso	Time (days) between collection date and reported symptoms date	numeric
age	Age of individual	numeric
sample_type	Type of sample	categorical
kit_test	Test kit used	categorical
laboratory	Laboratory performing the test	categorical
SARSCOV2	Test result	numeric

Model terms considered:

GAM term	Optional
s(time_pso,m=2)	no
s(time_pso,age,m=2,bs='fs')	yes
s(time_pso,sample_type,m=2,bs='fs')	yes
s(time_pso,kit_test,m=2,bs='fs')	yes
s(time_pso,laboratory,m=2,bs='fs')	yes

A total of 16 model variants are possible with the terms listed above. Models (model_structure) using a dataset Y were executed using the instruction:

bam(as.formula(model_structure), data=Y, family=binomial(link = 'logit'), nthreads=12, discrete = TRUE, control = gam.control(trace = F), select=TRUE)

The selected model variant was the first in the table below, where the two other best scoring models are presented:

Model structure	AIC difference to selected model
SARSCOV2 ~ s(time_pso,age,m=2,bs='fs')+ s(time_pso,kit_test,m=2,bs='fs')+ s(time_pso,laboratory,m=2,bs='fs')+ s(time_pso,m=2)+ s(time_pso,sample_type,m=2,bs='fs')	0
SARSCOV2 ~ s(time_pso,laboratory,m=2,bs='fs')+ s(time_pso,m=2)+ s(time_pso,sample_type,m=2,bs='fs')	-0.7121697
SARSCOV2 ~ s(time_pso,age,m=2,bs='fs')+ s(time_pso,laboratory,m=2,bs='fs')+ s(time_pso,m=2)+ s(time_pso,sample_type,m=2,bs='fs')	-0.7442026

The summary of the selected model:

```
Approximate significance of smooth terms:
                                edf Ref.df Chi.sq  p-value    
s(time_pso,age)           4.736e-06     25  0.000   0.9530    
s(time_pso,kit_test)      2.608e+00     54  5.230   0.0784 .  
s(time_pso,laboratory)    1.589e-05     41  0.000   0.5732    
s(time_pso)               9.909e-01      9 66.763   <2e-16 ***
s(time_pso,sample_type)   3.196e-01     24  0.458   0.2207    
---
Signif. codes:  0 '***' 0.001 '**' 0.01 '*' 0.05 '.' 0.1 ' ' 1
```

2) Odds ratios

For each dependent variable of interest X, we extracted the odds ratio from each selected GAM model (1, 2, 3.i, 3.ii, see above) by making model predictions over each unique value of X while using the mean / mode value (if numerical / categorical) of all other dependent variables. We then divided those predictions by the model prediction corresponding to a reference prediction, which was commonly defined as the mean / mode of the variable of interest (unless stated otherwise). See example in **Code example for model 3.ii**.

3) Code example for model 3.ii

The following code <model_3.ii.R> implements the model used for Figure 6B of the main text. It represents the smallest R script developed but it includes examples on how (1) models are run, (2) predictions are obtained, (3) odd ratios are obtained, (4) results are plotted. Note that a source file named "GAM_do_predictions_plus_odds.R" is used, the code of this file is included at the end.

Script <model_3.ii.R>

```
pacman::p_load(tidyverse, mgcv, extrafont, lubridate, gridExtra, oddsratio, ggplot2, cowplot)

### some functions

getmode<- function(X){
  t<- table(X)
  names(t)[which(t==max(t))]
}

### loading test data

##needs to be replaced by path to data file
load('datasets/data_swabs_mt1p_singlesd.Rdata') ##data_swabs_mt1p_singlesd
data<- data_swabs_mt1p_singlesd

##reject unknown values of variables of interest
data <- data %>%
  filter(laboratory!="Unknown")   %>%
  filter(kit_test!="Unknown")

##restrict time since symptoms to 100 days
data<- data %>% filter(time_pso<100)

##build a time series of the positive rate
data_positive_rate<- data %>% group_by(time_pso) %>% summarise(N = n(), positive_rate = sum(SARSCOV2, na.rm = T) / N)  %>% ungroup() %>%
as.data.frame()

##select only time steps that have N>=10 samples
data_positive_rate<- data_positive_rate %>% filter(N>9)
```

```
selected_time_pso<- data_positive_rate$time_pso
data<- data %>% filter(time_pso %in% selected_time_pso)

##count how many patients had their last test = pos
##save those that actually had their last test = neg to create a subdataset for this model
count<- 0
not_last_pos_patient<- c()
for(pat in unique(data$patient_uID)){
  thispat<- data[which(data$patient_uID==pat),] ##get this patient
  thispat<- thispat[order(thispat$date_collection),] ##order by test date
  all_test_result<- thispat$SARSCOV2 ##get test results
  if(tail(all_test_result,1)==1) count<- count +1 ##if last test is POS, count
  else not_last_pos_patient<- c(not_last_pos_patient, pat)
}
freq_last_was_positive<- count/length(unique(data$patient_uID))
print(paste("frequency of individuals with last test positive = ", freq_last_was_positive))

##this is the subdataset of those that actually have their last test = neg
data_not_last_positive<- data %>% filter(patient_uID %in% not_last_pos_patient)

##this is the positive rate of the subdataset
data_positive_rate_special<- data_not_last_positive %>% group_by(time_pso) %>% summarise(N = n(), positive_rate = sum(SARSCOV2, na.rm = T) / N)  %>%
ungroup() %>% as.data.frame()

### run the model

model_structure<- "SARSCOV2 ~ s(time_pso,kit_test,m=2,bs='fs') + s(time_pso,m=2)"
model <- bam(as.formula(model_structure), data=data_not_last_positive, family=binomial(link = 'logit'), nthreads=12, discrete = TRUE, control =
gam.control(trace = F), select=TRUE)
AIC(model) ##check AIC
summary(model) ##summarize model

### model prediction based on time_pso (all other variables with most common value)

predictions_frame<- expand.grid(time_pso = 0:60, ##range to present in output
                                kit_test= getmode(data_not_last_positive$kit_test)) ##get most common kit_test

predictions_exclude<- c("s(kit_test)") ##tell predict.gam to ignore kit_test in predictions_frame

##set some variables for the shared code within "source file" below
model<- model
doOdds<- FALSE ##do not calculate odds, since all variables excluded in prediction
refcat<-"" ##category reference for odds
z_variable<- "" ##categories for odds
x_variable<- "" ##x variable for odds
trans<- plogis ##to transform from link function in model
source("GAM_do_predictions_plus_odds.R") #predictions_frame, predOdds_time, predOdds_cats
data_odds_time_time<- predOdds_x
data_odds_cats_time<- predOdds_z
data_predict_time<- predictions_frame

### model prediction based on kit_test

predictions_frame<- expand.grid(time_pso = 0:60,
                              kit_test= unique(data_not_last_positive$kit_test))

predictions_exclude<- c() ##tell predict.gam to include all variables in predictions_frame

##set some variables for the shared code within "source file" below
model<- model
doOdds<- TRUE ##calc odds between categories in kit_test
refcat<- getmode(data$kit_test) ##category reference for odds
z_variable<- "kit_test" ##categories for odds
x_variable<- "time_pso" ##x variable for odds
trans<- plogis ##to transform from link function in model
source("GAM_do_predictions_plus_odds.R") #predictions_frame, predOdds_time, predOdds_cats
data_odds_time_kit<- predOdds_x
data_odds_cats_kit<- predOdds_z
data_predict_kit<- predictions_frame

dataplot<- data_predict_time %>% filter(time_pso<= 45)
dataplot<- dataplot %>% select(time_pso,lower,upper,fit) %>% mutate(type1='model', type2="model")
dataplot<- dataplot %>% bind_rows( data_positive_rate_special %>% mutate(time_group=time_pso) %>% select(time_pso,positive_rate) %>%
rename(fit=positive_rate) %>% mutate(lower=NA, upper=NA, type1="data", type2="data") )
gtime<- ggplot()+ theme_classic() + geom_ribbon(data=dataplot %>% filter(type2=="model"),aes(x=time_pso, ymin = lower, ymax = upper, group=type1),
fill='grey', alpha=0.5)+ geom_jitter(data=data_not_last_positive,aes(x=time_pso,y=SARSCOV2, col='data'), height = 0, inherit.aes = F, pch = "|", width= 1, size=1,
alpha = 1)+ geom_line(data=dataplot %>% filter(type2=="model"),aes(x=time_pso, y=fit, color="model"), size=1) +
ggtitle("Data & model prediction (time only)") + ylim(0,1) + xlab("time since symptoms (days)") + ylab("probability of test positive") +
theme(legend.position="right") + scale_color_manual("", values=c("tomato","black"), labels=c("data","model"))
```

```
dataplot<- data_predict_kit %>% filter(!is.na(data_predict_kit$kit_test)) %>% mutate(kit_test=as.character(kit_test))
dataplot<- dataplot %>% select(time_pso,lower,upper,fit,kit_test) %>% mutate(type1=kit_test, type2="model")
dataplot<- dataplot %>% bind_rows( data_positive_rate_special %>% select(time_pso,positive_rate) %>% rename(fit=positive_rate) %>% mutate(kit_test=NA,
lower=NA, upper=NA, type1="data", type2="data") )
gkit<- ggplot()+ theme_classic() + geom_ribbon(data=dataplot %>% filter(type2=="model"),aes(x=time_pso, ymin = lower, ymax = upper, group=type1, fill=type1),
alpha=0.2)+ geom_line(data=dataplot %>% filter(type2=="model"),aes(x=time_pso, y=fit, color=type1), size=1) +
geom_jitter(data=data_not_last_positive,aes(x=time_pso,y=SARSCOV2), height = 0, inherit.aes = F, pch = "|", width= 1, col='tomato3', size=1, alpha = 1)+
ggtitle("Data & model prediction (by kit & time)") + ylim(0,1) + xlim(0,45) + theme(legend.position=c(0.5,0.95)) +
theme(legend.position="right",legend.key.width = unit(1.3, 'cm'),legend.key.height = unit(0, 'cm')) +
guides(color = guide_legend("", ncol = 1),fill = guide_legend("", ncol = 1)) + xlab("time since symptoms (days)") + ylab("probability of test positive")

print(plot_grid(gtime, gkit, labels=LETTERS[1:2], ncol=2))
```

Which code should result in the following figure.

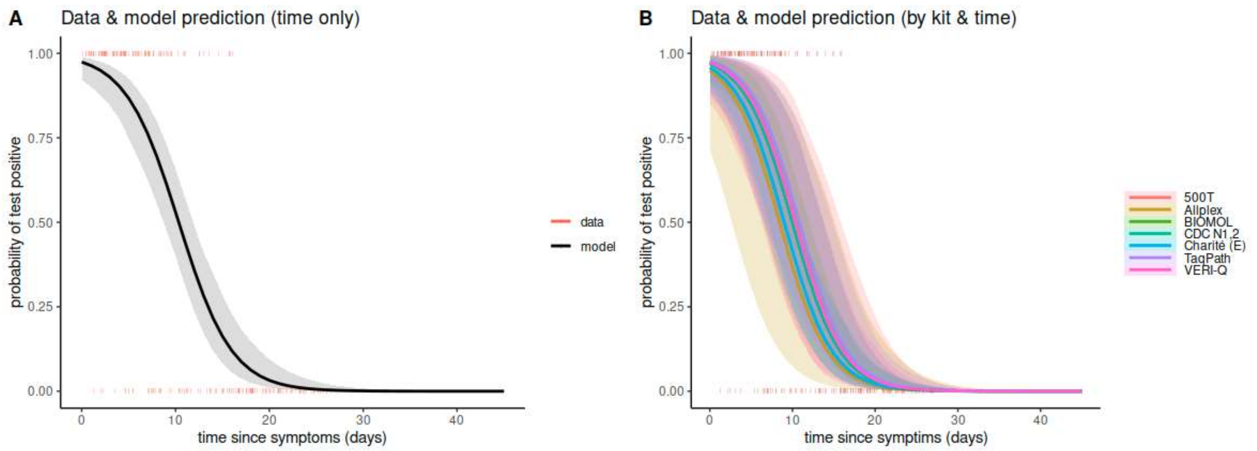

Script <GAM_do_predictions_plus_odds.R>

```
##make predictions calling predict.gam
print("<< calculating predictions >>")
checkPreds <- predict.gam(model, newdata = predictions_frame, exclude = predictions_exclude, type = "lpmatrix")

## extract parameters
beta <- coef(model)
## posterior mean and cov of coefs
Vb <- vcov(model, unconditional = TRUE)
## set 100 predictions to be executed
n <- 100
br <- MASS::mvrnorm(n, beta, Vb) # draw params from model posterior
predSims <- matrix(NA, nrow = n, ncol = nrow(checkPreds))
for (i in 1:n) { ## loop to get estimated effects for each param draw
  predSims[i, ] <- checkPreds %*% br[i,]  ## curve for each prediction replicate
}

## build a predictions data frame for output
predictions_frame<- predictions_frame  %>%
                  mutate(fit = trans(colMeans(predSims)),
                        upper = trans(as.numeric(apply(predSims, 2, quantile, prob = .975, type = 8))),
                        lower = trans(as.numeric(apply(predSims, 2, quantile, prob = .025, type = 8))))

## calculate the odds across all unique values / categories of the desired variable
predOdds_x<- c()
predOdds_z<- c()
## cicle time and variable values / categories and calc odds ratios
if(doOdds){
  print("<< calculating odds >>")
  #### brute force division by prediction for the reference value / category
  predSims4Odds<- data.frame(p=plogis(t(predSims)),x=predictions_frame[[x_variable]], z=predictions_frame[[z_variable]])
  predOdds<- data.frame(matrix(0, nrow=dim(t(predSims))[1], ncol=dim(t(predSims))[2]),x=predictions_frame[[x_variable]], z=predictions_frame[[z_variable]])
  start_time <- Sys.time()
  for(tt in unique(predSims4Odds$x)){
    td<- predSims4Odds %>% filter(x==tt)
    trd<- td %>% filter(z==refcat) ##reference category
    for(cat in unique(predSims4Odds$z)){
      print(paste(x_variable,tt,"--",z_variable,cat))
      odds<- as.numeric( (td %>% filter(z==cat))[1:n] ) / as.numeric( trd[1:n] )
      predOdds[which(predOdds$x==tt & predOdds$z==cat),1:n]<- odds
    }
```

```
  }
  ## build a human friendly / tidy data.frame
  predOdds<- predOdds %>% gather("pred", "value", -x, -z)
  predOdds_x<- predOdds %>% group_by(x, z) %>%
summarize(oddsme=mean(value),upper=quantile(value,probs=c(0.975),na.rm=TRUE),lower=quantile(value,probs=c(0.025),na.rm=TRUE)) %>% data.frame
  predOdds_z<- predOdds %>% group_by(z) %>%
summarize(oddsme=mean(value),upper=quantile(value,probs=c(0.975),na.rm=TRUE),lower=quantile(value,probs=c(0.025),na.rm=TRUE)) %>% data.frame
  end_time <- Sys.time()
  print(end_time - start_time)
  rm(predSims4Odds) ## release memory
}
```
