# Supplementary material for: Dynamics and Determinants of SARS-CoV-2 RT-PCR Testing on Symptomatic Individuals Attending Healthcare Centers during 2020 in Bahia, Brazil
Source: Viruses. 2022 Jul 15;14(7):1549. doi: 10.3390/v14071549 (PMC9321627; doi:10.3390/v14071549)
Supplement: Supplementary file 1 [file viruses-14-01549-s001.zip › File S2-PCR Bahia BR V4 VIRUSES ISSUE - material.pdf]

Dynamics and determinants of SARS-CoV-2 RT-PCR testing on symptomatic individuals attending healthcare centers during 2020 in Bahia, Brazil.

Supplementary Tables
Supplementary Figures
Supplementary References

Table S1 - test kits present in the dataset

Assay (name, details)	ID / Acronym
“Allplex 2019-nCoV Assay” from Seegene. The assay targets three viral targets: the E gene (specific of the subgenus Sarbecovirus), the N and the RdRP genes (both specifics of the SARS-CoV-2) [1]	Allplex
“KIT BIOMOL OneStep/COVID-19” from Hybribrio (China) and Paraná Institute of Molecular Biology (IBMP, Brazil). The assay targets the conserved region ORF 1ab and the N gene [1,2]	BIOMOL
“Protocolo CDC Vírus Respiratórios”, which targets the N1 and N2 nucleocapsid protein gene regions [3]	CDC N1,2
“Protocolo CDC SARS-CoV2 (N1, N2 e N3)”, targeting the N1, N2 and N3 nucleocapsid protein gene regions [4]	CDC N1,2,3
“Protocolo Charité SARS-CoV2 (E)” from Charité Universitätsmedizin Berlin Institute of Virology) which targets the E gene region [2,5,6]; Protocolo Charité: SARS-CoV2 (E/P1) from Charité Universitätsmedizin Berlin Institute of Virology) which targets the E gene region [2,5,6]; Protocolo Charité: SARS-CoV2 (E/RP) from Charité Universitätsmedizin Berlin Institute of Virology) which targets the E gene region [2,5,6]	Charité (E)
“2019 NCOV 500T” by Integrated DNA Technologies (IDT) targeting the S gene region [7]	500T IDT
“TaqPath COVID 19 CE-IVD” by Applied Biosystems, which targets the ORF-1ab, spike (S) protein and nucleocapsid (N) protein regions [8]	TaqPath
“VERI-Q nCoV-QS” by MiCo-BioMed that targets the ORF and N genes [9]	VERI-Q

Table S2 - public laboratories present in the dataset

Full name	**ID / Acronym**
Laboratório central de saúde pública professor gonçalo moniz	LCDSPPGM
Laboratório estadual de referência regional	LEDRR
Laboratório municipal de referência regional de porto seguro	LMDRRDPS
Laboratório municipal de referência regional de paulo afonso	LMDRRDPA
Laboratório municipal de referência regional de vitória da conquista	LMDRRDVDC
Centro de pesquisas gonçalo muniz	CDPGM
Laboratório de imunologia do instituto de ciência da saúde	LDIDIDCDS
Laboratório estudos de agentes infecciosos e vetores	LEDAIEV
Laboratório de farmacogenética e epidemiologia molecular	LDFEEM

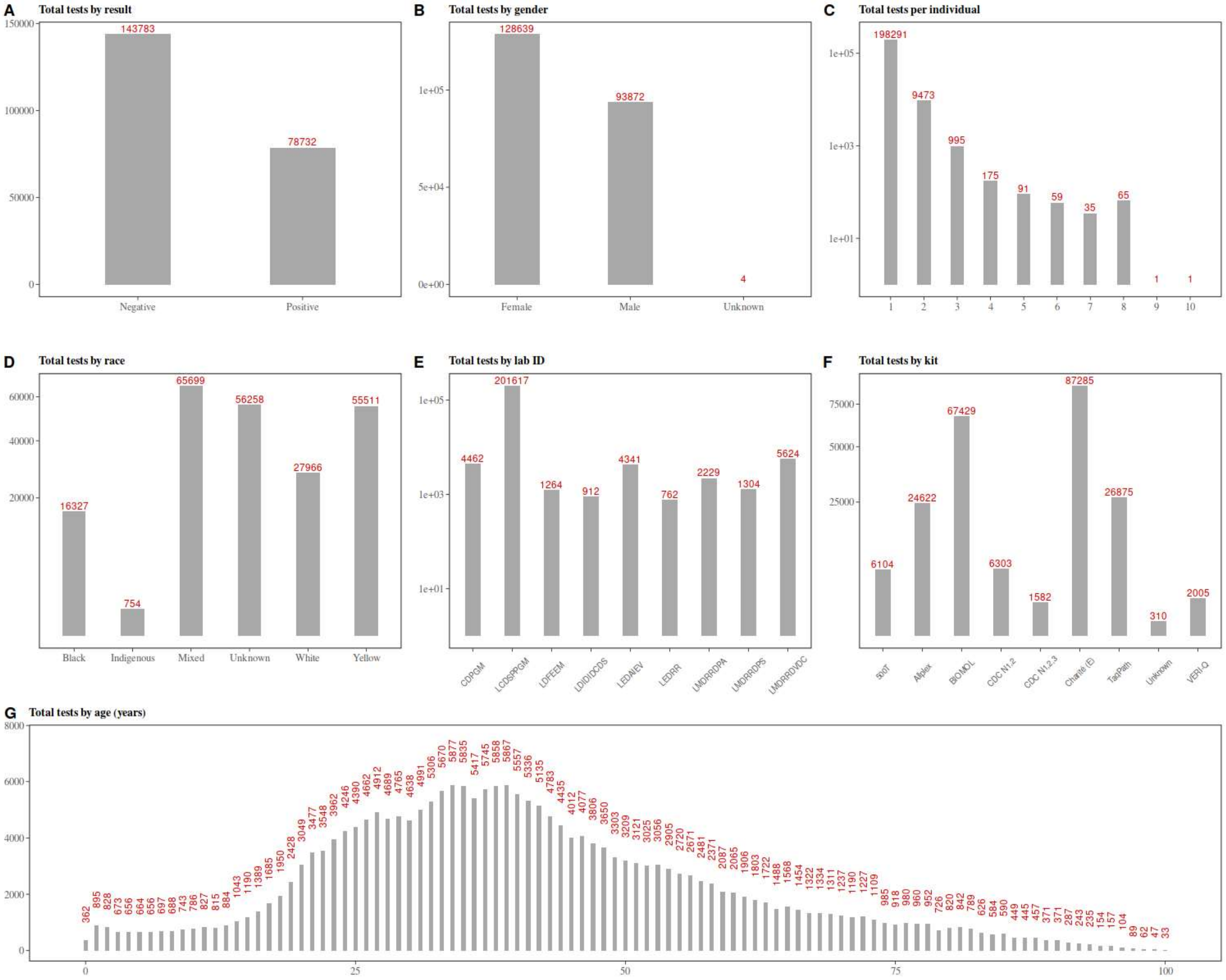

Supplementary Figure S1 - number of tests according to several variables. Panels show the number of tests by result (positive and negative) (A), reported gender (B), number of repeat tests per unique individual (C), reported race (D), laboratory (E), test kit (F) and age (G). Kit tests are identified by unique ID (see **Table S1**). Laboratories are identified by unique ID (see **Table S2**). Race follows the official classifications. Ages are binned in 1 year categories and trimmed to 100.

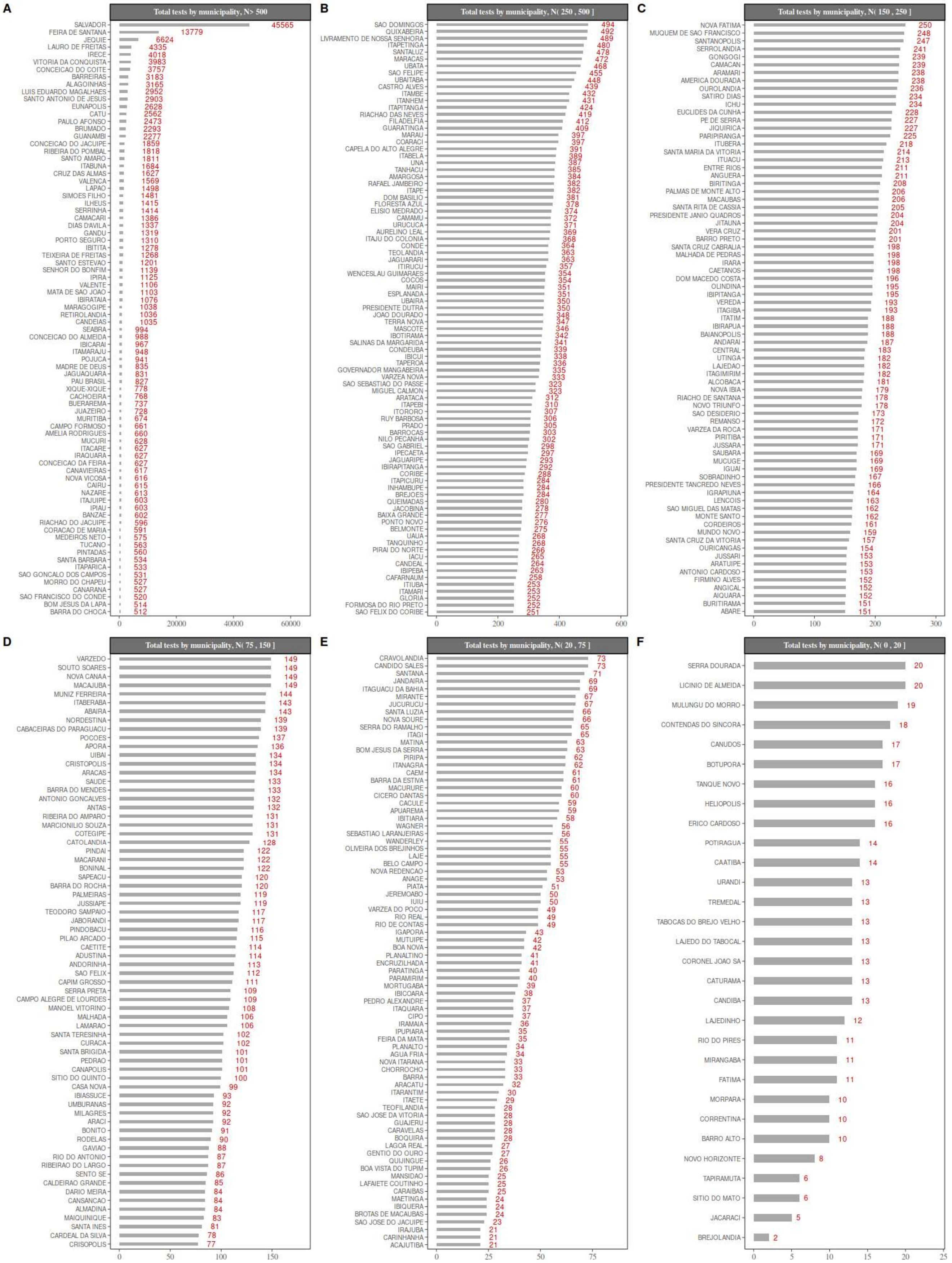

Supplementary Figure S2 - number of tests in each municipio of Bahia. For visualisation purposes, different panels present the number of tests (in red) per municipio under different thresholds.

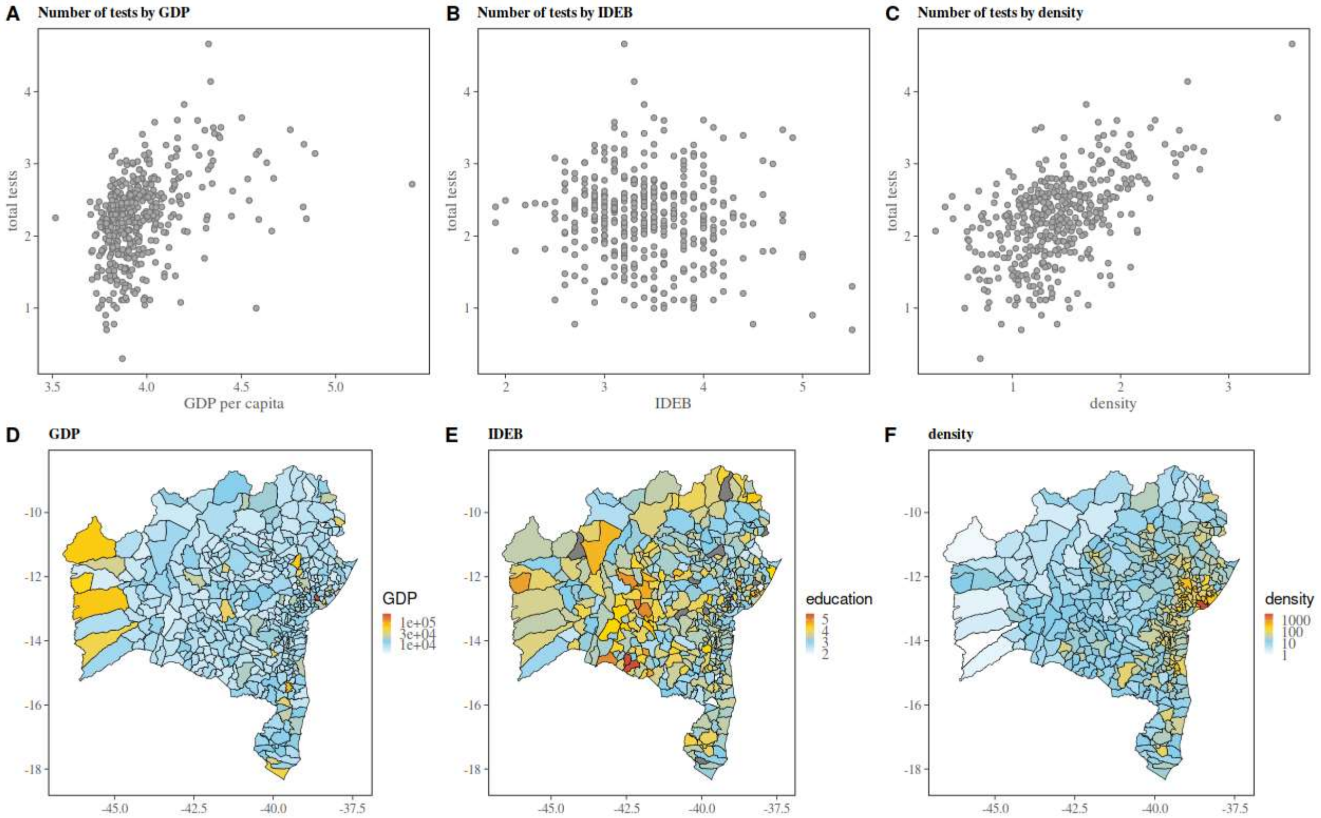

Supplementary Figure S3 - relationships of socio-demographic variables and number of tests in dataset. (A-C) Variables: GDP = GDP per capita, IDEB = education index, density = hab/Km^2 (see main text for full description of variables and sources). Variables GDP, density, tests are presented with log10 transformation. **(D-F)** Mapping of variables in the subpanels A-C. "education" stands for IDEB

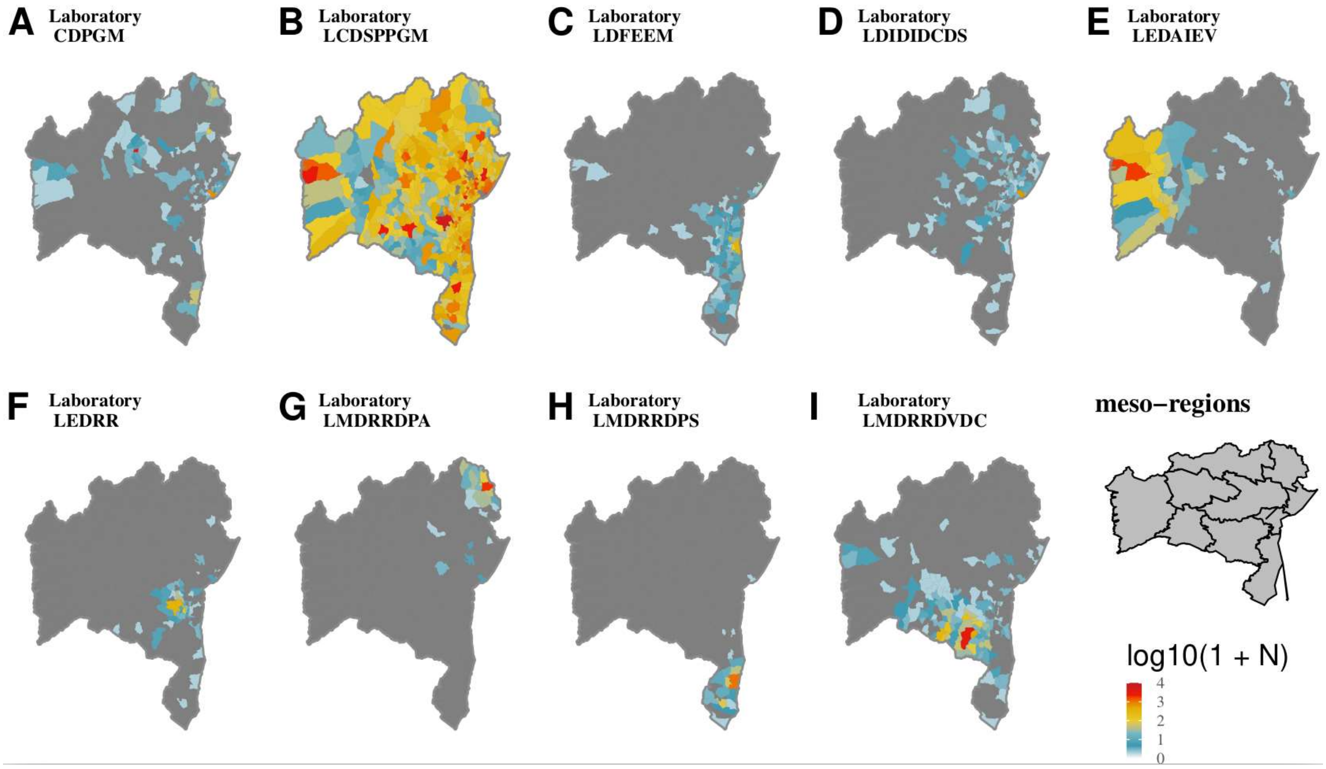

Supplementary Figure S4 - number of tests per municipality per laboratory. Each panel is the geographical range (catchment) of each laboratory, coloured according to the number of tests performed in the observation period (shown after transformation with log10 +1). The laboratory LCDSPPGM is the only laboratory for which catchment is across the entire state of Bahia. The final panel presents the official borders of the higher order spatial dimension termed meso-regions.

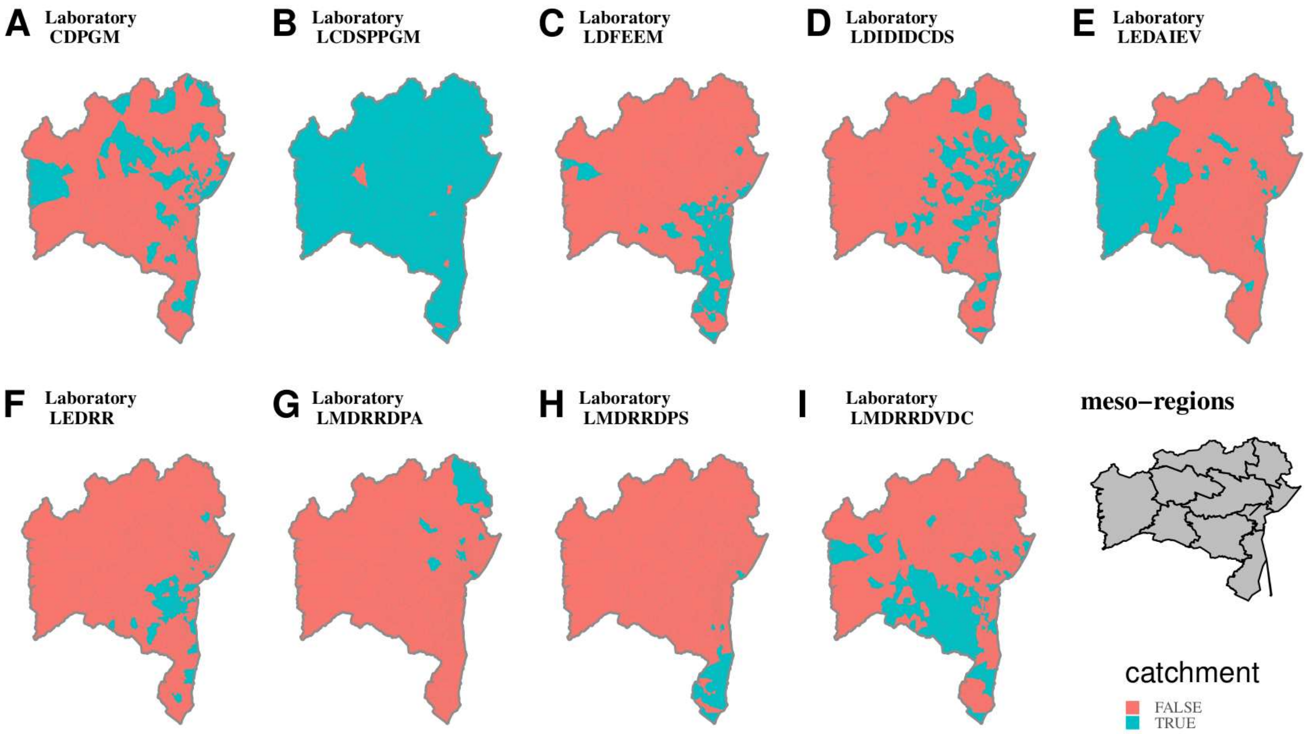

Supplementary Figure S5 - catchment area for each laboratory. Each panel is the geographical range (catchment) of each laboratory, coloured according to whether the laboratory had tests in each municipality: TRUE if yes, FALSE if no. The laboratory LCDSPPGM is the only laboratory for which catchment is across the entire state of Bahia. The final panel presents the official borders of the higher order spatial dimension termed meso-regions.

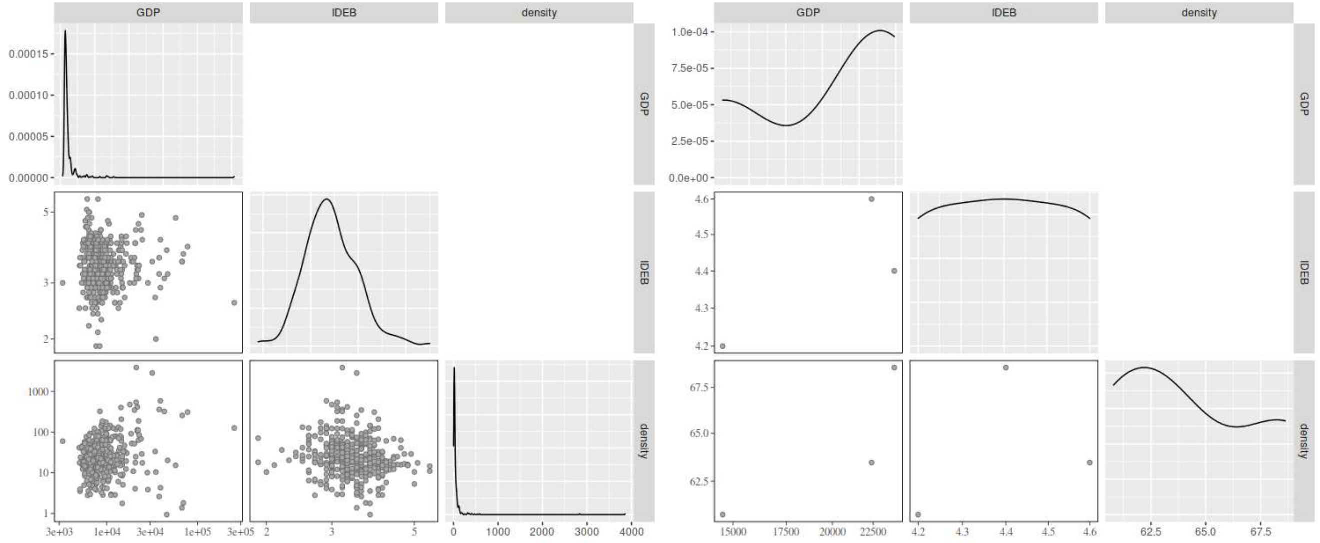

Supplementary Figure S6 - Relationships of socio-demographic variables. (left) GDP versus IDEB and density for the sub dataset (ii) used in the results of **Figures 4-5** of the main text. **(right)** Same plot as on the left panel but restricting density > 50, GDP > 10000 and IDEB > 4, showing that only 3 municipalities exist with a combination of high values of the three variables. (**both**) GDP = GDP per capita, IDEB = education index, density = hab/km^2. X and Y axes are transformed to log10 for visualisation.

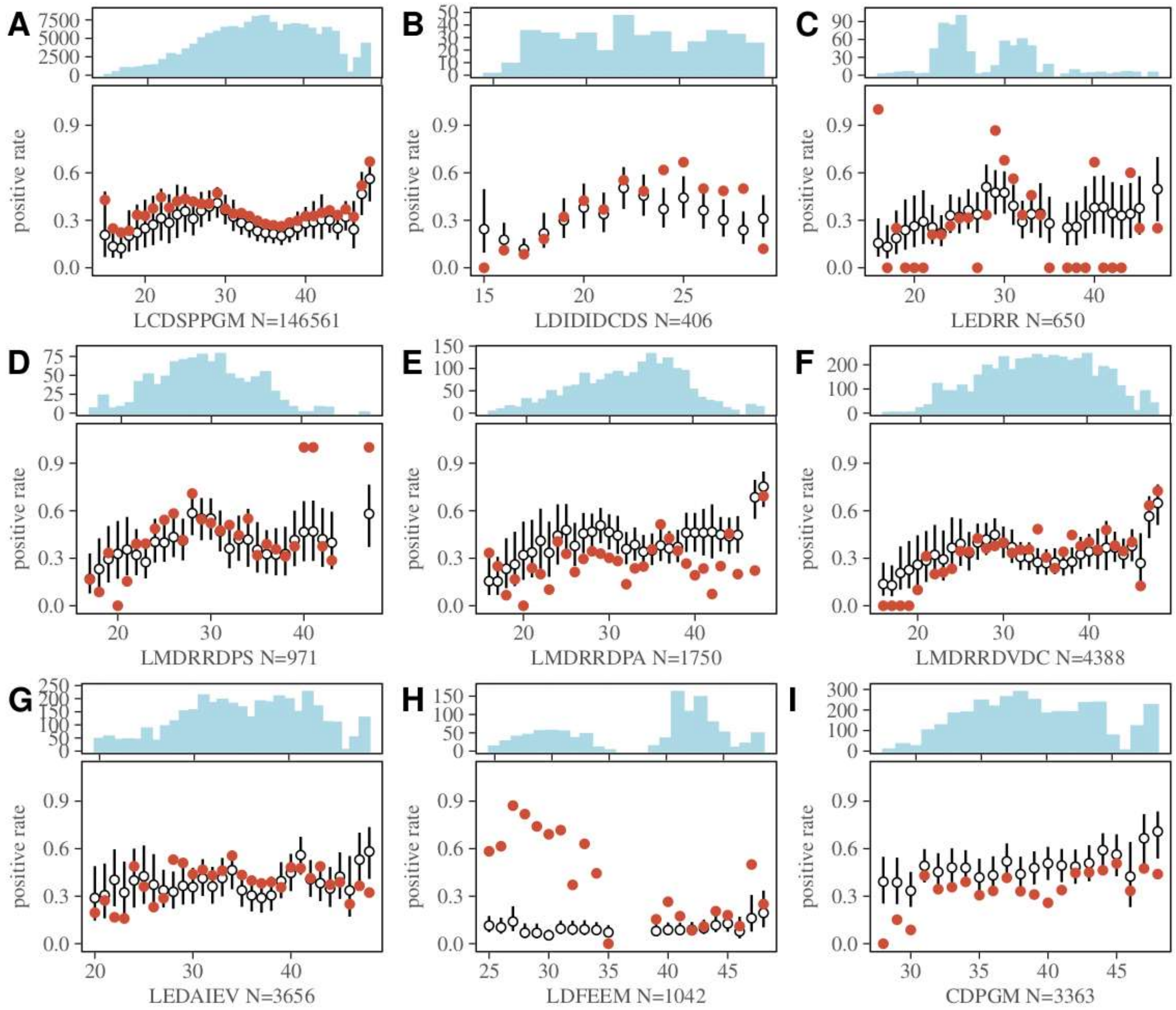

Supplementary Figure S7 - output of Model 1 per laboratory (positive rate). Each panel is the model's output per laboratory (named on the x-axis, along with the number of total tests performed). The top subpanels are the number of tests per week (blue bars). The bottom subpanels are the model output (open white circles) versus the data (red full circles) in terms of positive rate per week. This figure complements the results of Figure 4 in the main text.

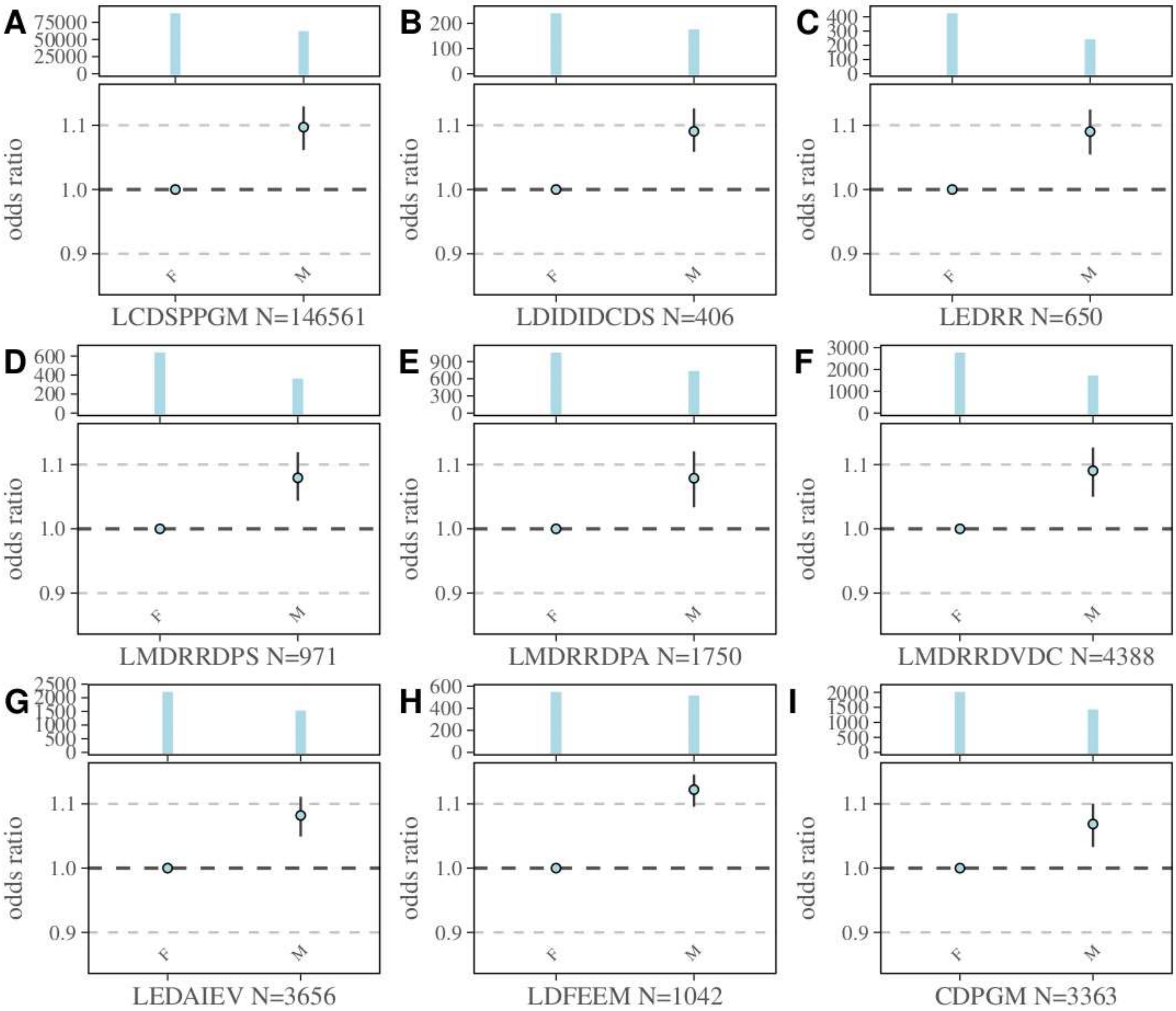

Supplementary Figure S8 - output of Model 1 per laboratory (gender OR for positive rate). Each panel is the model's output per laboratory (named on the x-axis, along with the number of total tests performed). The top subpanels are the number of tests per week according to gender (blue bars). The bottom subpanels are the model output in terms of the odds ratios (OR) for positive rate according to gender (odds of getting a positive test) (see **Supplementary Text 1** for details). This figure complements the results of Figure 4 in the main text.

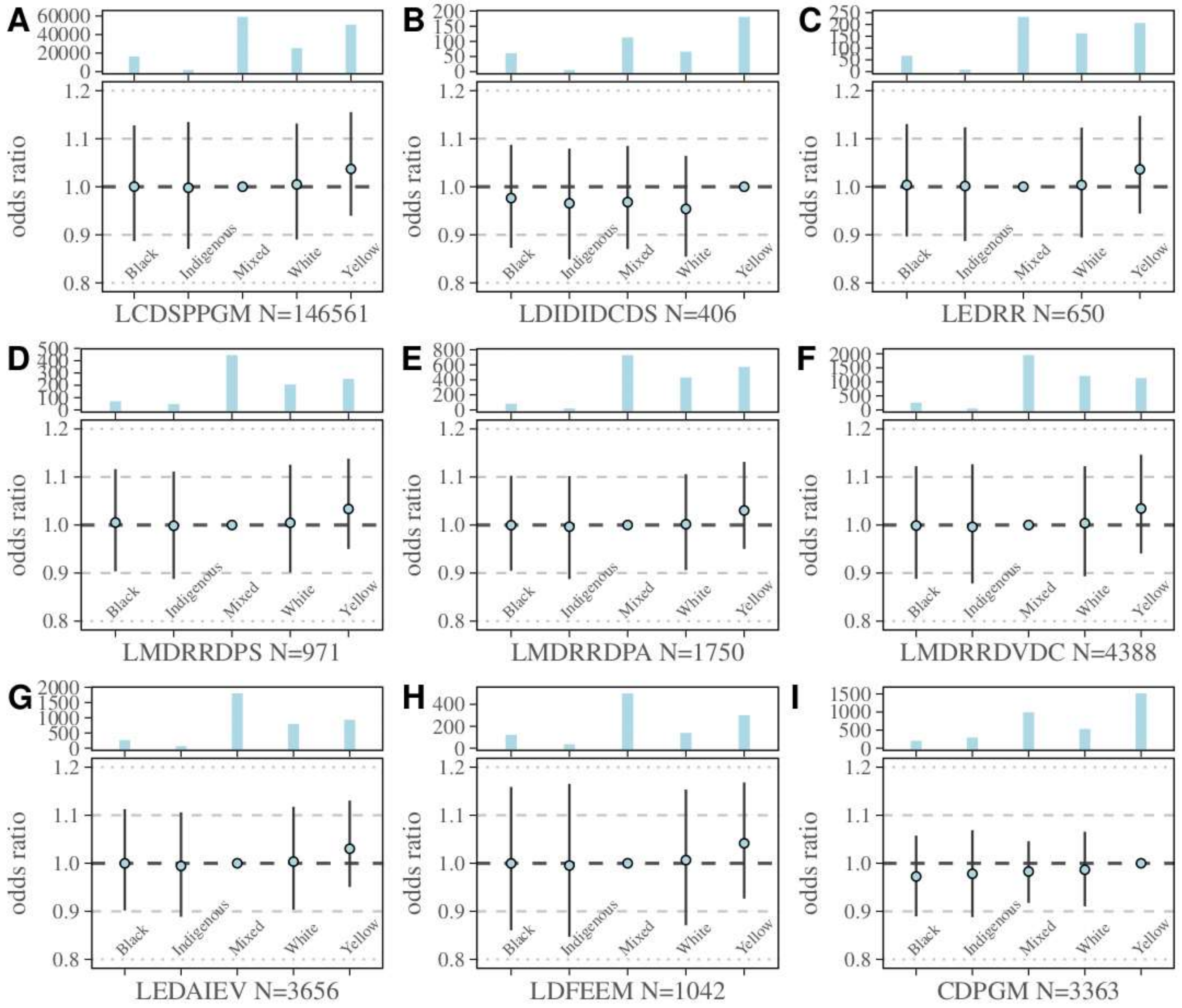

Supplementary Figure S9 - output of Model 1 per laboratory (race OR for positive rate). Each panel is the model's output per laboratory (named on the x-axis, along with the number of total tests performed). The top subpanels are the number of tests per week according to race (blue bars). The bottom subpanels are the model output in terms of the odds ratios (OR) for positive rate according to race (odds of getting a positive test) (see **Supplementary Text 1** for details). This figure complements the results of Figure 4 in the main text.

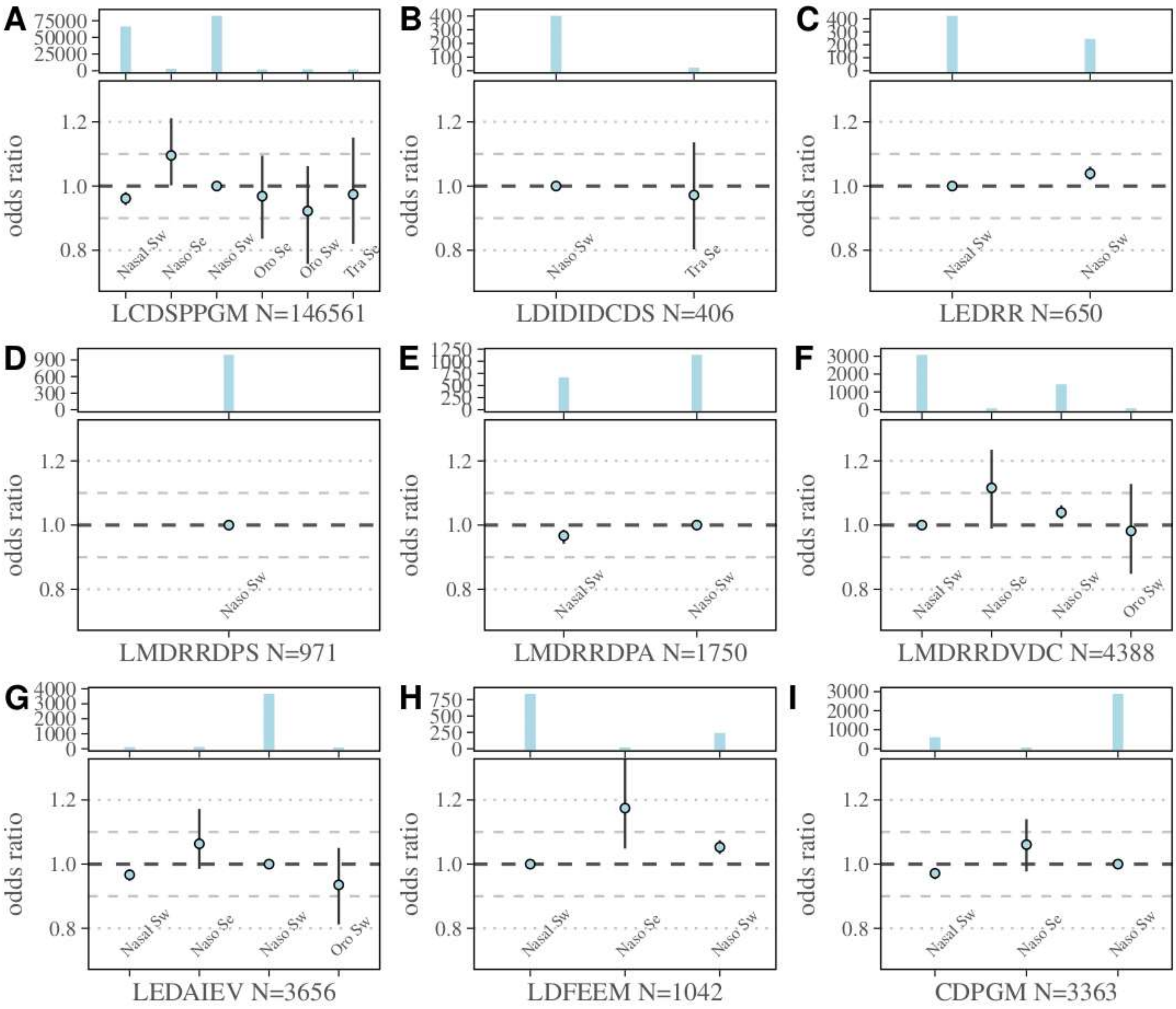

Supplementary Figure S10 - output of Model 1 per laboratory (sample type OR for positive rate). Each panel is the model's output per laboratory (named on the x-axis, along with the number of total tests performed). The top subpanels are the number of tests per week according to sample type (blue bars). The bottom subpanels are the model output in terms of the odds ratios (OR) for positive rate according to sample type (odds of getting a positive test) (see **Supplementary Text 1** for details). Sample type key: "Naso Sw" = nasopharyngeal swab, "Oro Se" = oropharyngeal secretion, "Naso Se" = nasopharyngeal secretion, "Oro Sw" = oropharyngeal swab, "Tra Se" = tracheal secretion, "Nasal Sw" = nasal swab. This figure complements the results of Figure 4 in the main text.

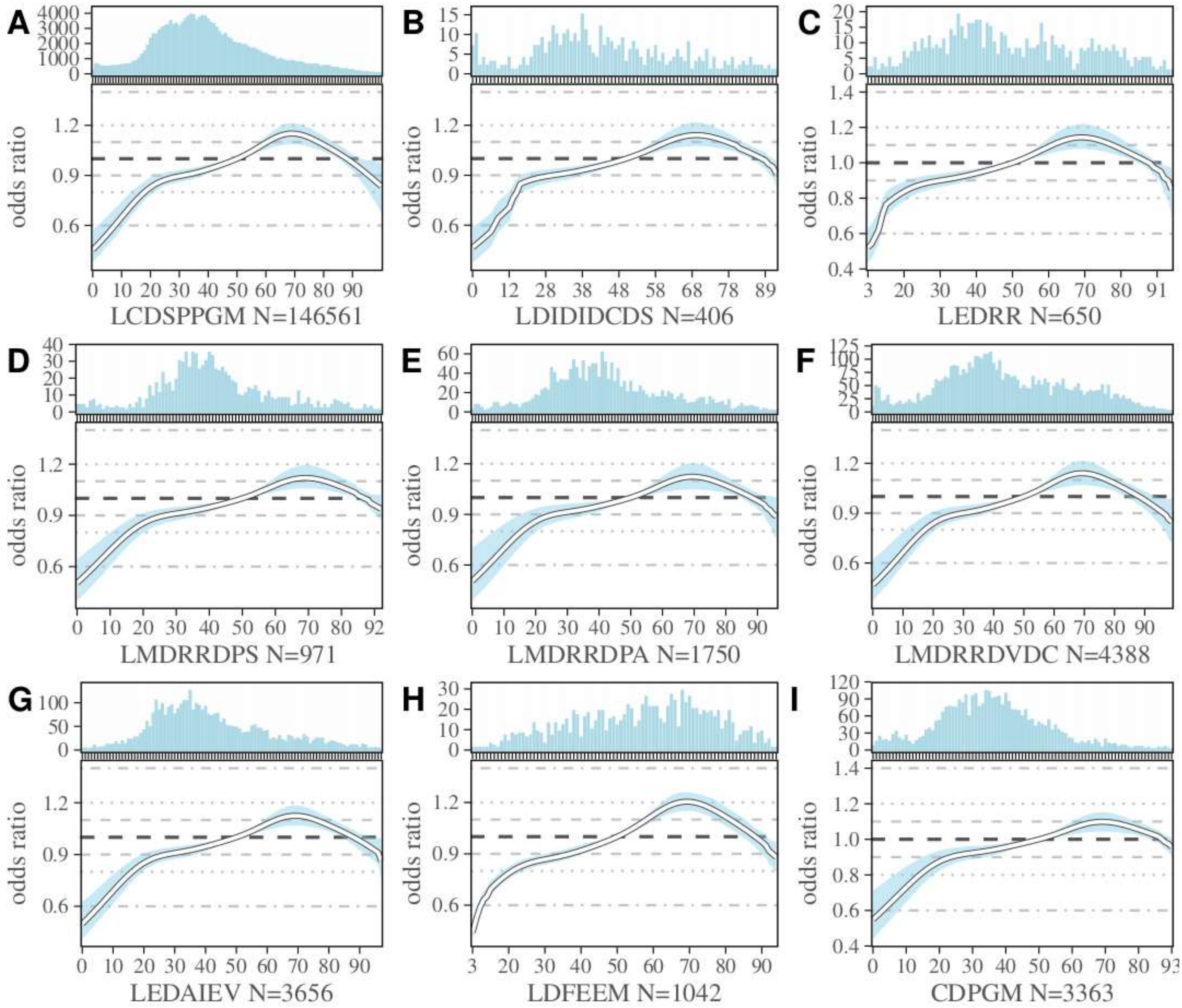

Supplementary Figure S11 - output of Model 1 per laboratory (age OR for positive rate). Each panel is the model's output per laboratory (named on the x-axis, along with the number of total tests performed). The top subpanels are the number of tests per week according to age (blue bars). The bottom subpanels are the model output in terms of the odds ratios (OR) for positive rate according to age (odds of getting a positive test) (see **Supplementary Text 1** for details). This figure complements the results of Figure 4 in the main text.

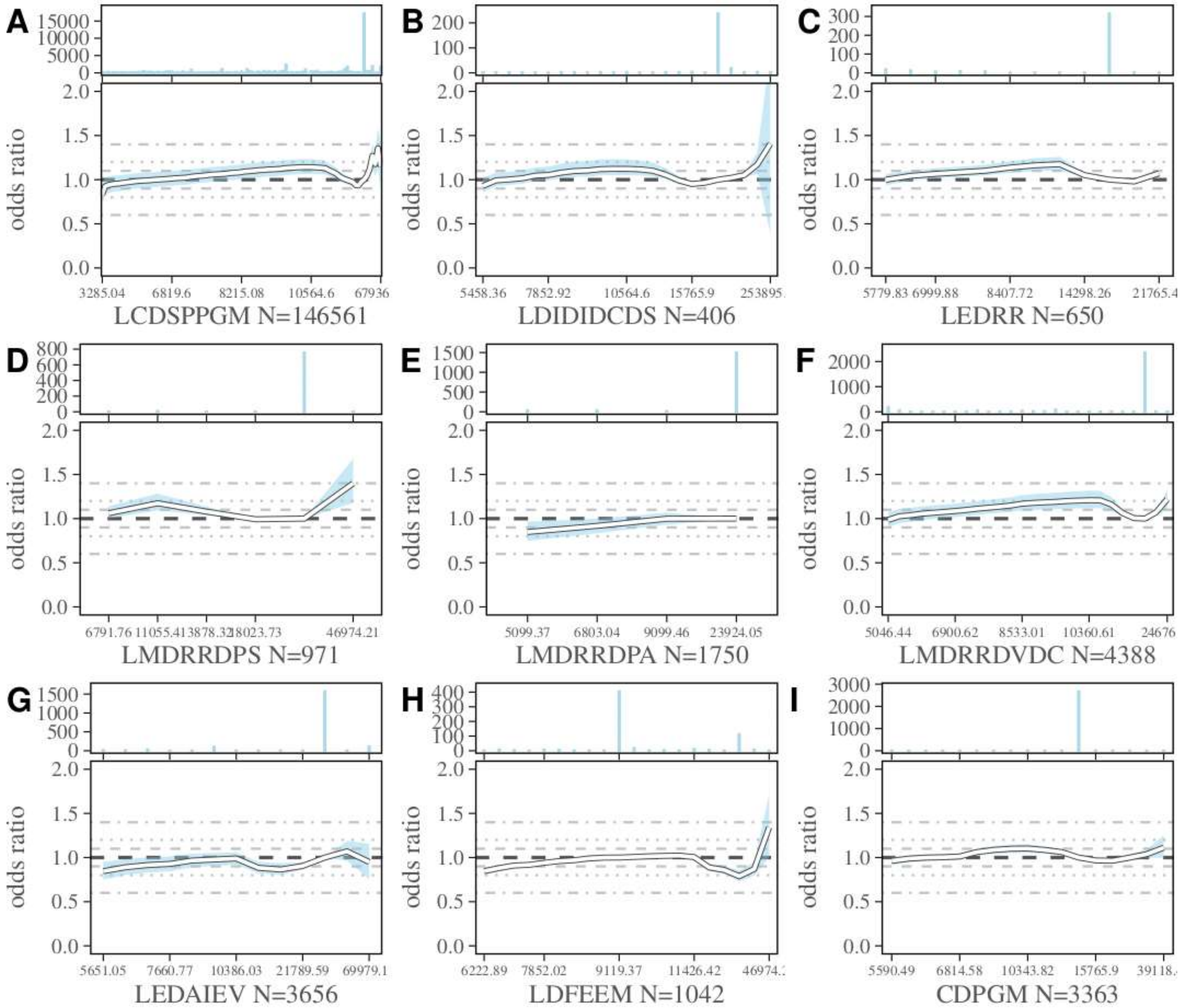

Supplementary Figure S12 - output of Model 1 per laboratory (GDP OR for positive rate). Each panel is the model's output per laboratory (named on the x-axis, along with the number of total tests performed). The top subpanels are the number of tests per week according to GDP of municipality (blue bars). The bottom subpanels are the model output in terms of the odds ratios (OR) for positive rate according to GDP of municipality (odds of getting a positive test) (see **Supplementary Text 1** for details). This figure complements the results of Figure 4 in the main text.

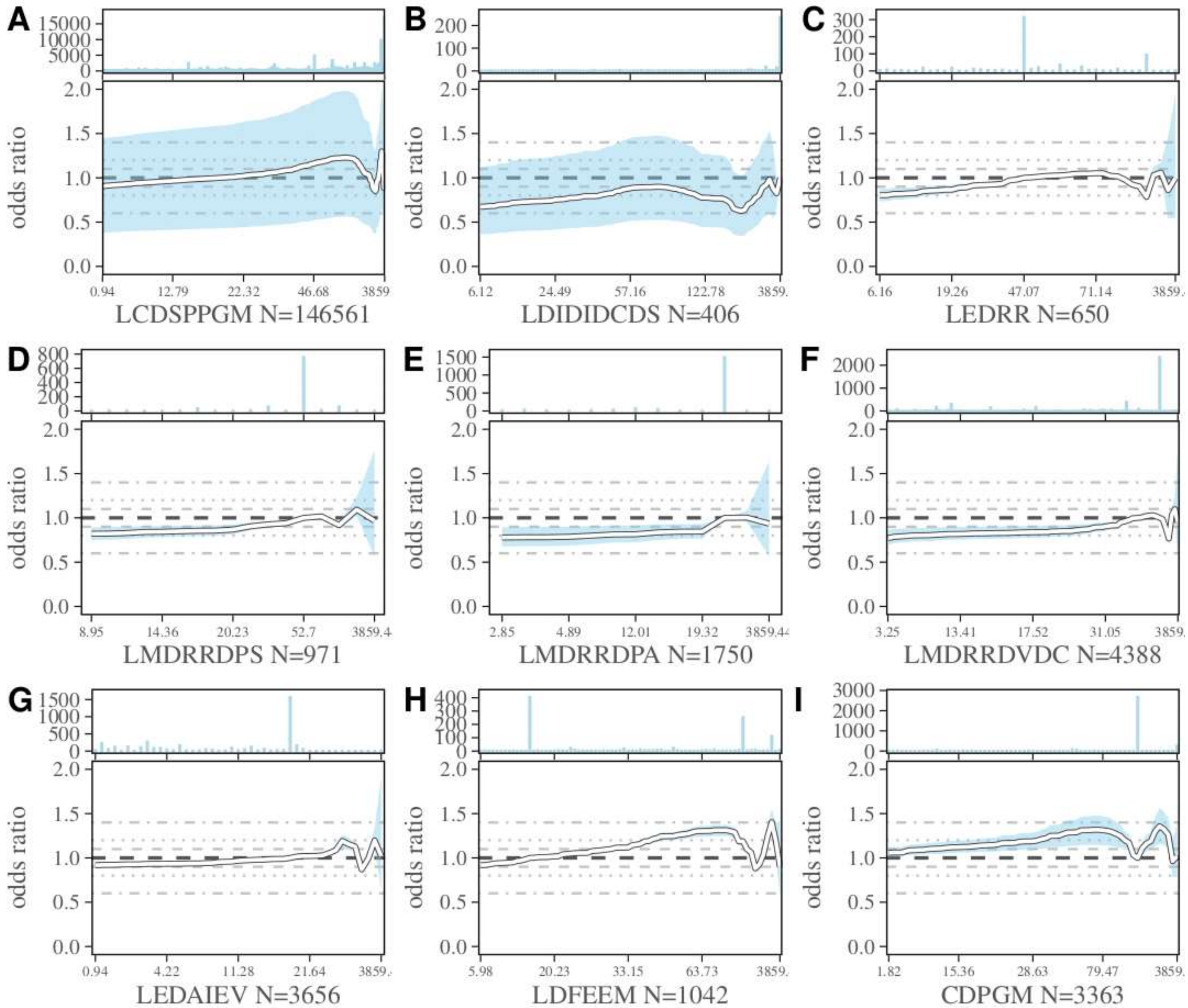

Supplementary Figure S13 - output of Model 1 per laboratory (density OR for positive rate). Each panel is the model's output per laboratory (named on the x-axis, along with the number of total tests performed). The top subpanels are the number of tests per week according to density of municipality (blue bars). The bottom subpanels are the model output in terms of the odds ratios (OR) for positive rate according to density of municipality (odds of getting a positive test) (see **Supplementary Text 1** for details). This figure complements the results of Figure 4 in the main text.

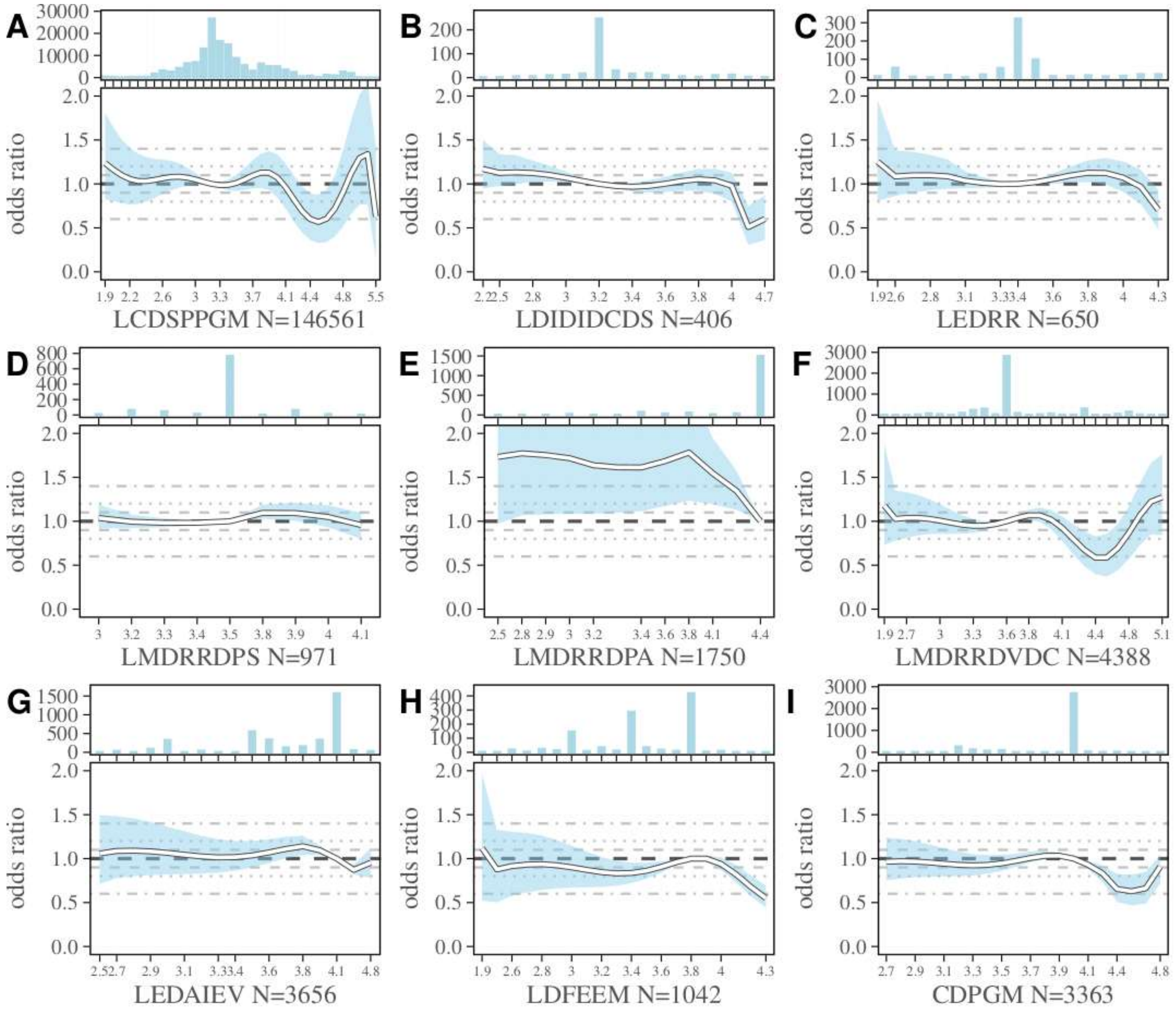

Supplementary Figure S14 - output of Model 1 per laboratory (IDEB OR for positive rate). Each panel is the model's output per laboratory (named on the x-axis, along with the number of total tests performed). The top subpanels are the number of tests per week according to IDEB of municipality (blue bars). The bottom subpanels are the model output in terms of the odds ratios (OR) for positive rate according to IDEB of municipality (odds of getting a positive test) (see **Supplementary Text 1** for details). This figure complements the results of Figure 4 in the main text.

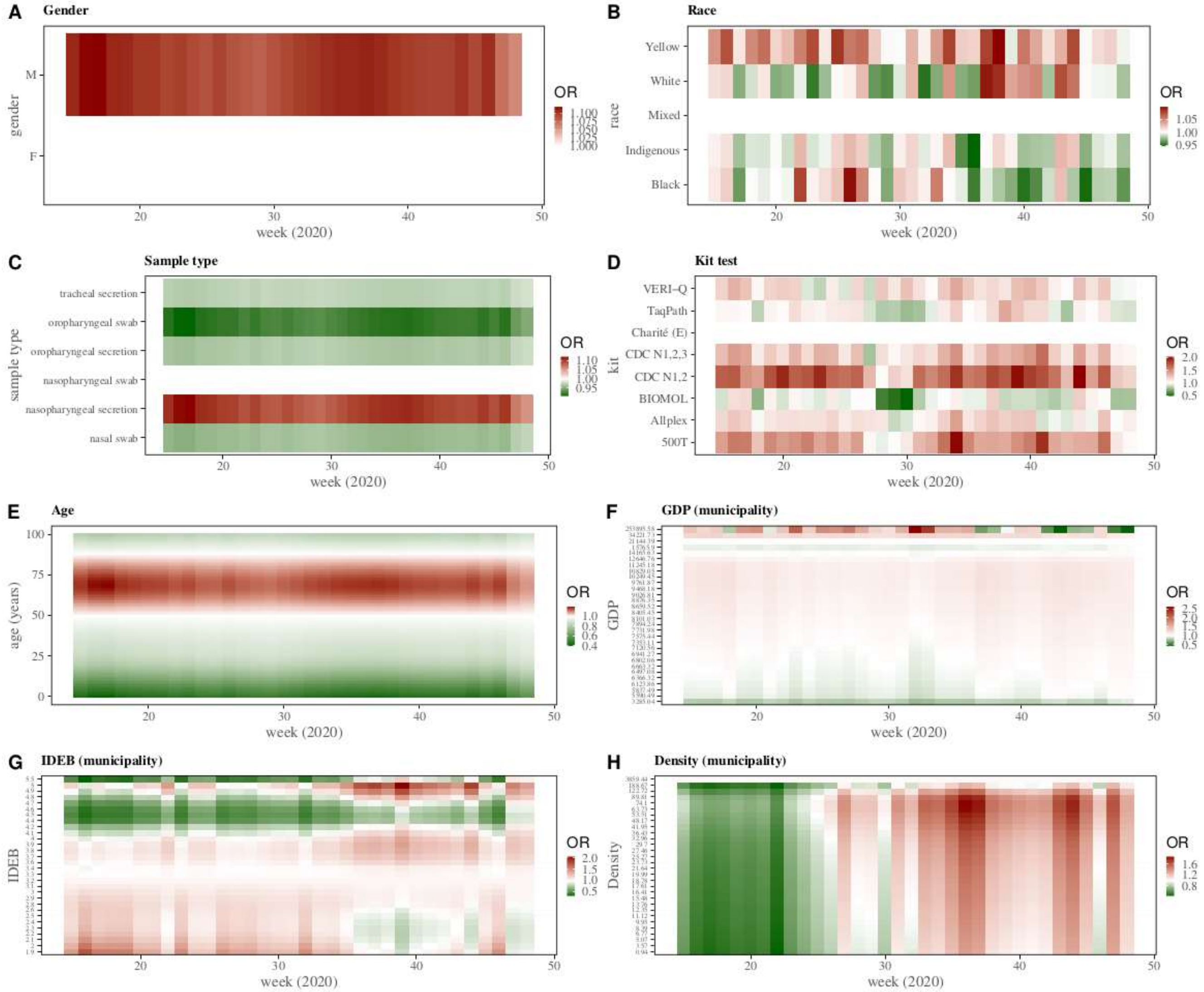

Supplementary Figure S15 - OR output of Model 1 per week per variable. Mean of odds ratio (OR) per week, per gender **(A)**, race **(B)**, sample type **(C)**, kit test **(D)**, age **(E)**, GDP of municipality **(F)**, IDEB of municipality **(G)**, density of municipality **(H)**. In all subpanels, an OR = 1 is set to white; any odds above one will be in different shades of red, and below one in shades of green. For each variable, the most common value / category is set as reference such that its OR = 1 (white) (except age, set at 50 years). Note that for age and GDP, the y-axis ticks are subsampled for visualisation (given the highly non-linear nature of the variables in this dataset). This figure complements the results of Figure 4 in the main text.

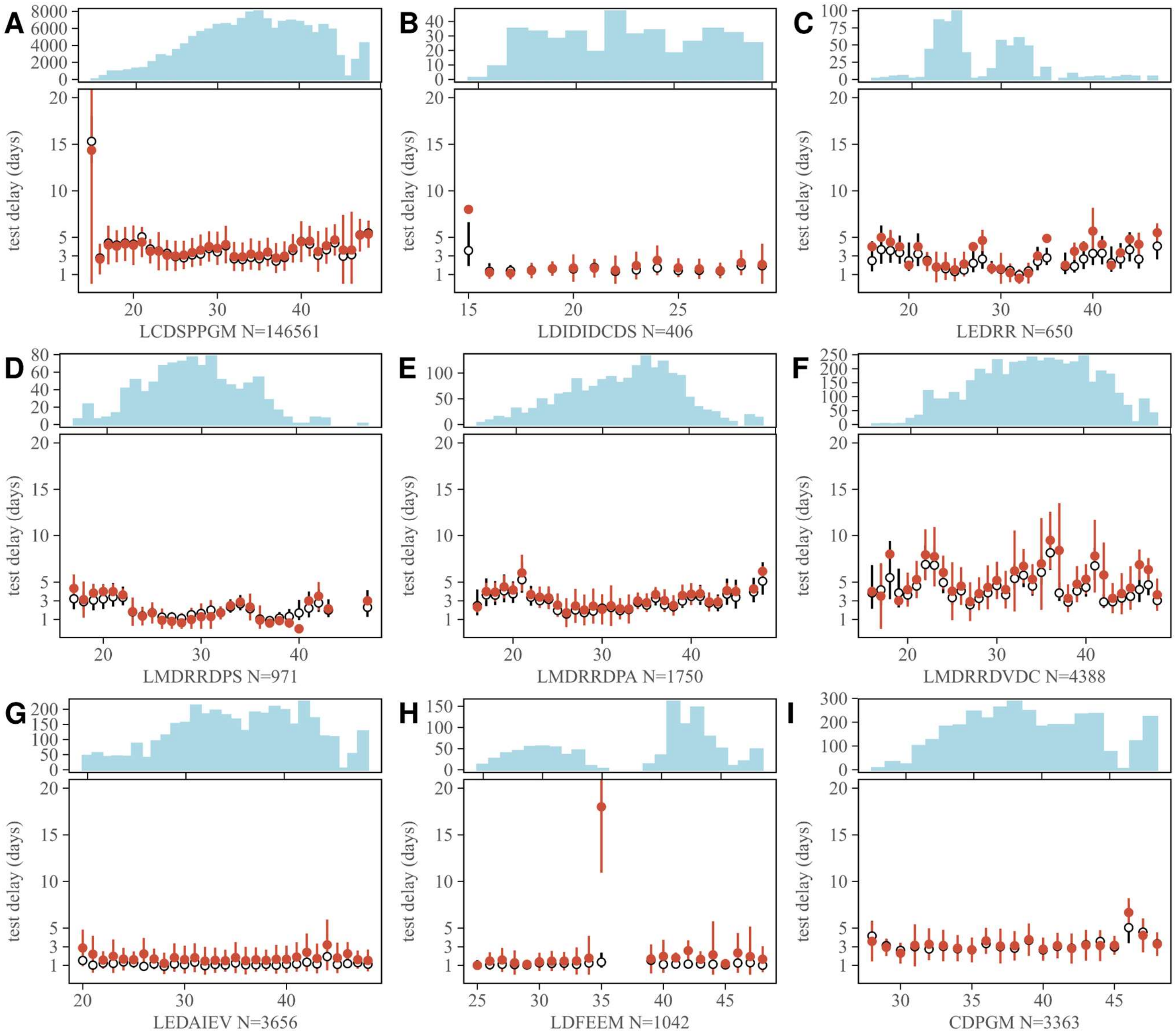

Supplementary Figure S16 - output of Model 2 per laboratory (test delay). Each panel is the model's output per laboratory (named on the x-axis, along with the number of total tests performed). The top subpanels are the number of tests per week (blue bars). The bottom subpanels are the model output (open white circles) versus the data (red full circles) in terms of test delay per week. This figure complements the results of Figure 5 in the main text.

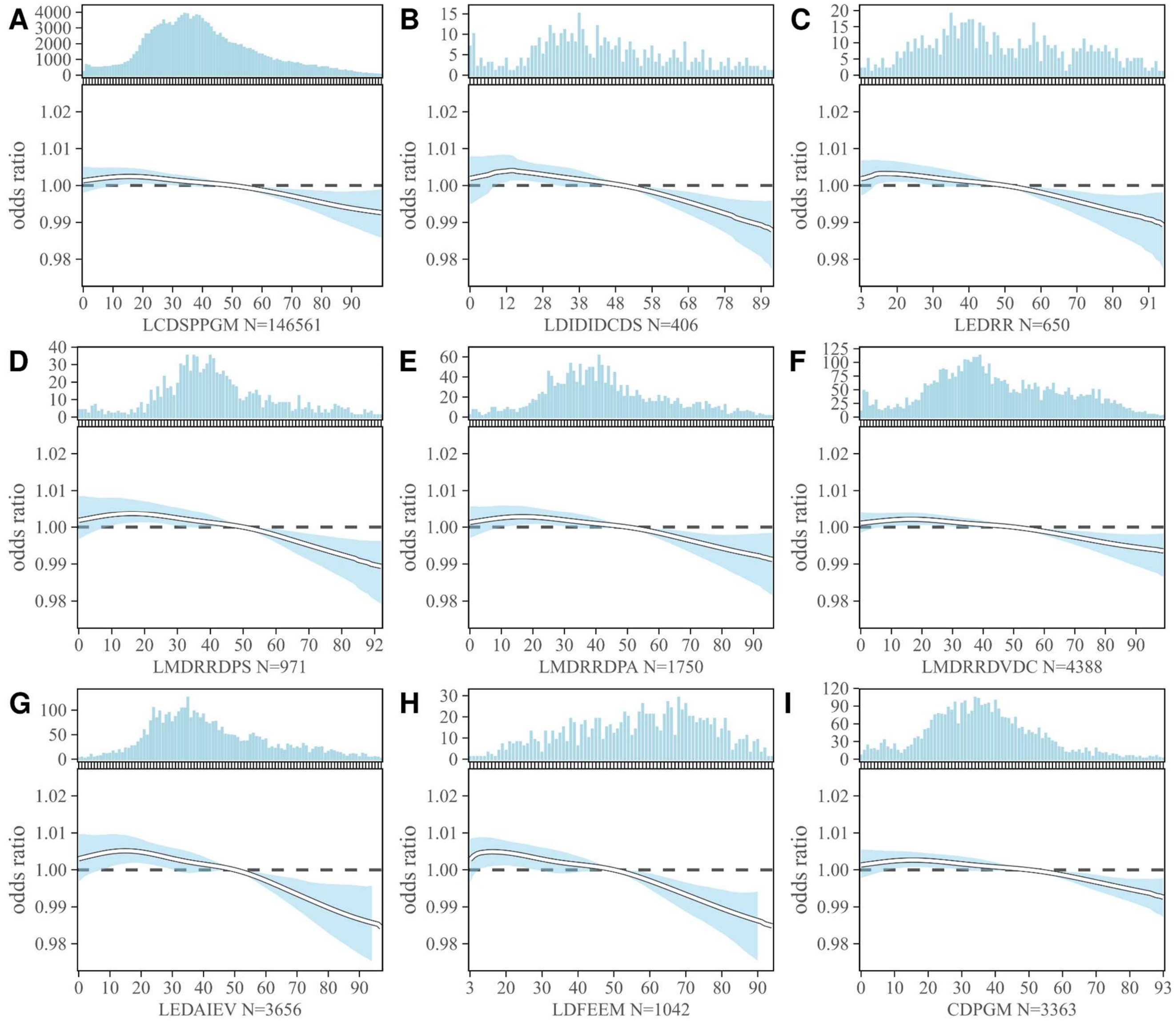

Supplementary Figure S17 - output of Model 2 per laboratory (age OR for positive rate). Each panel is the model's output per laboratory (named on the x-axis, along with the number of total tests performed). The top subpanels are the number of tests per week according to age (blue bars). The bottom subpanels are the model output in terms of the odds ratios (OR) for positive rate according to age (odds of getting a positive test) (see **Supplementary Text 1** for details). This figure complements the results of Figure 5 in the main text.

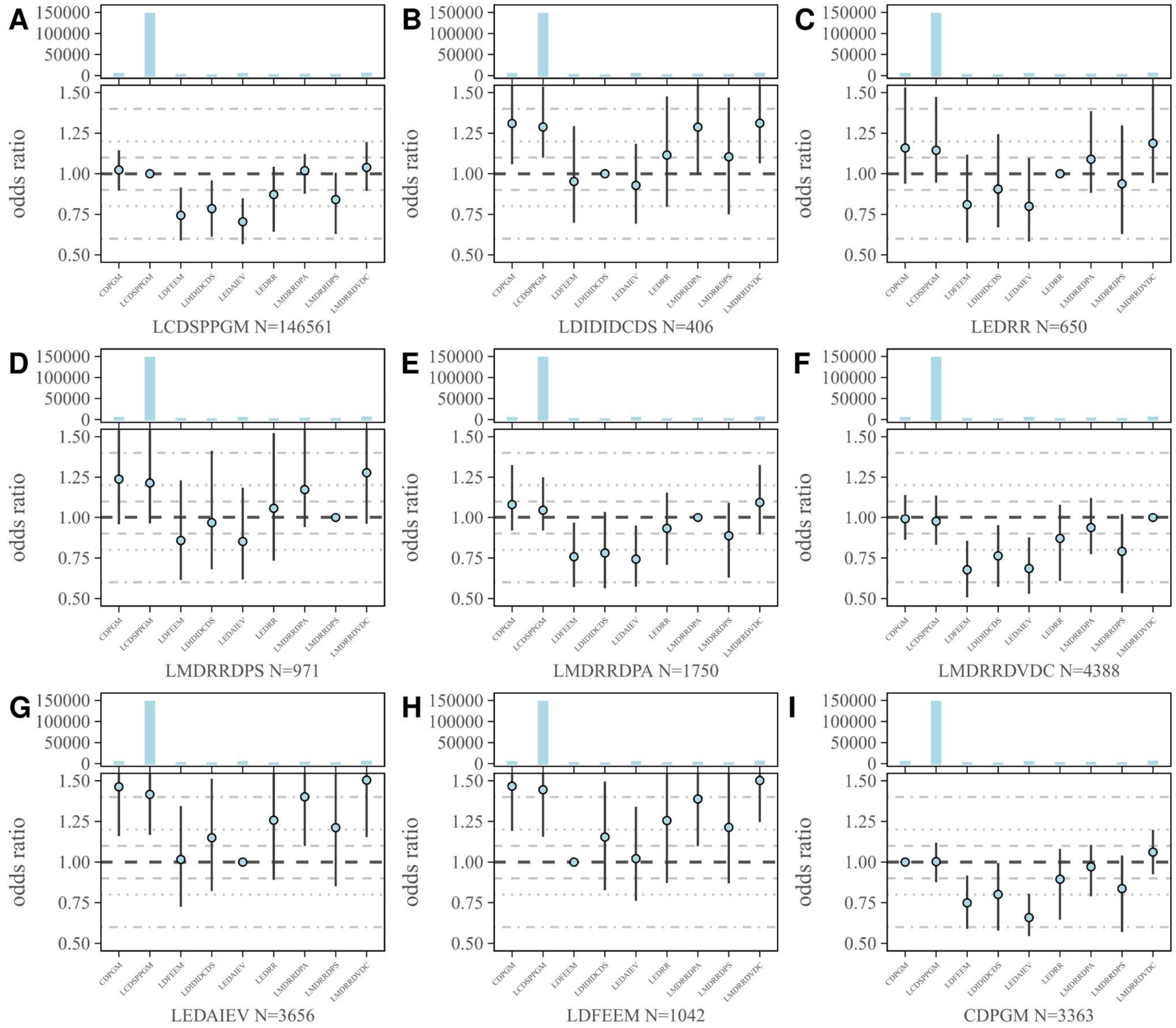

Supplementary Figure S18 - output of Model 2 per laboratory (laboratory OR for positive rate). Each panel is the model's output per laboratory (named on the x-axis, along with the number of total tests performed). The top subpanels are the number of tests per week according to laboratory (blue bars). The bottom subpanels are the model output in terms of the odds ratios (OR) for positive rate according to laboratory (odds of getting a positive test) (see **Supplementary Text 1** for details). This figure complements the results of Figure 5 in the main text.

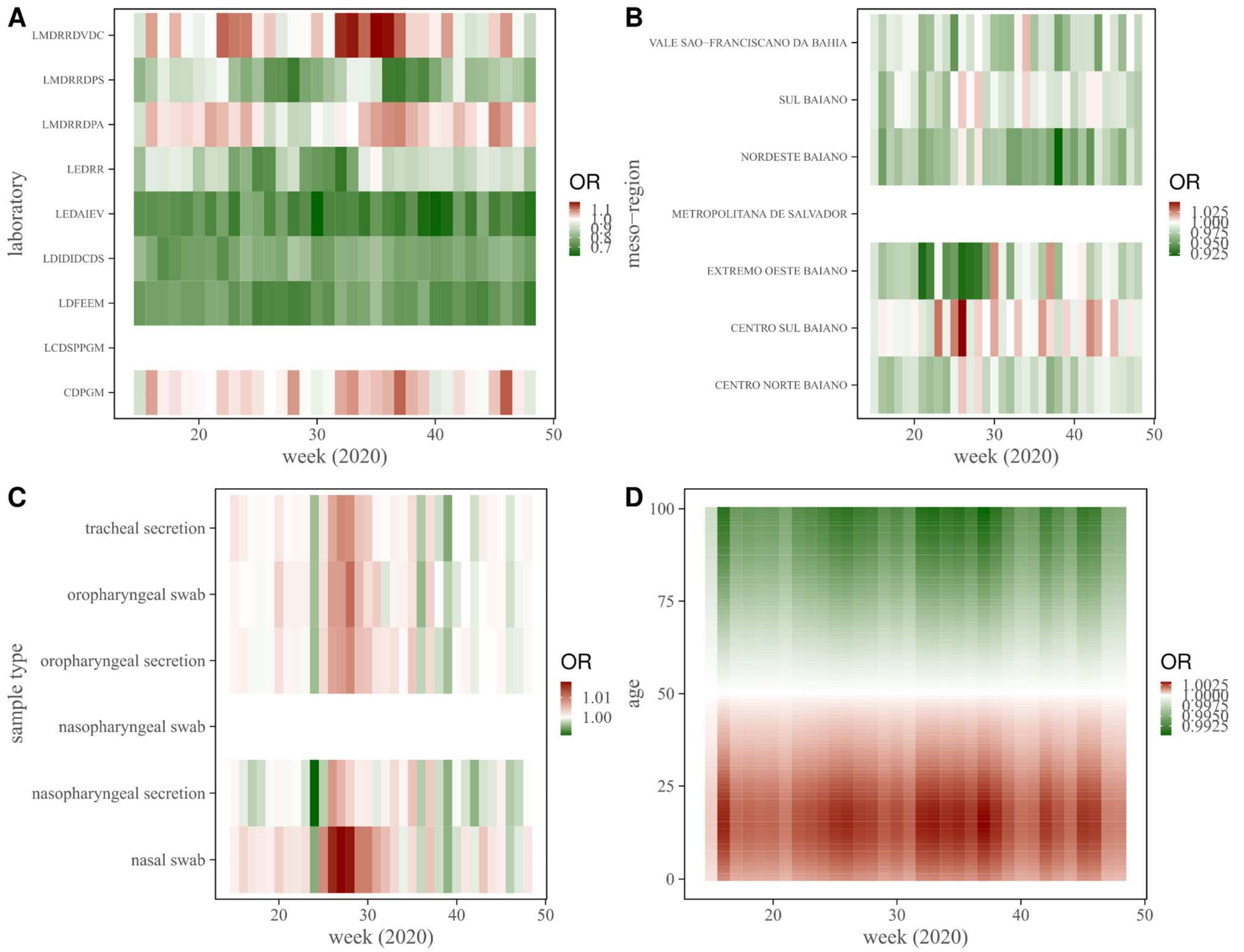

Supplementary Figure S19 - OR output of Model 2 per week per variable. Mean of odds ratio (OR) per week, per laboratory **(A)**, meso-region **(B)**, sample type **(C)**, age **(D)**. In all subpanels, an OR = 1 is set to white; any odds above one will be in different shades of red, and below one in shades of green. For each variable, the most common value / category is set as reference such that its OR = 1 (white) (except age, set at 50 years). This figure complements the results of Figure 5 in the main text.

References

1. Farfour E, Lesprit P, Visseaux B, Pascreau T, Jolly E, Houhou N, et al. The Allplex 2019-nCoV (Seegene) assay: which performances are for SARS-CoV-2 infection diagnosis? Eur J Clin Microbiol Infect Dis. 2020;39: 1997–2000.

2. Corman VM, Landt O, Kaiser M, Molenkamp R, Meijer A, Chu DKW, et al. Detection of 2019 novel coronavirus (2019-nCoV) by real-time RT-PCR. Eurosurveillance. 2020. doi:10.2807/1560-7917.es.2020.25.3.2000045

3. CDC 2019-Novel Coronavirus (2019-nCoV) Real-Time RT-PCR Diagnostic Panel. [cited 10 Nov 2020]. Available: https://www.fda.gov/media/134922/download

4. Lu X, Wang L, Sakthivel SK, Whitaker B, Murray J, Kamili S, et al. US CDC Real-Time Reverse Transcription PCR Panel for Detection of Severe Acute Respiratory Syndrome Coronavirus 2. Emerg Infect Dis. 2020;26. doi:10.3201/eid2608.201246

5. WHO - Diagnostic detection of 2019-nCoV by real-time RT-PCR. [cited 10 Nov 2020]. Available: https://www.who.int/docs/default-source/coronaviruse/protocol-v2-1.pdf

6. BIO-manguinhos - Kit molecular SARS-CoV-2 (in Portuguese). [cited 10 Nov 2020]. Available: https://www.bio.fiocruz.br/images/molec-sars-cov2-e-rp-rox-2x48r-11-05-2020-lotes-01ao05.pdf

7. FDA - Linea COVID-19 Assay Kit. [cited 10 Nov 2020]. Available: https://www.fda.gov/media/138059/download

8. Applied Biosystems TaqPath COVID-19 CE-IVD RT-PCR Kit. [cited 10 Nov 2020]. Available: https://aslm.org/wp-content/uploads/2020/05/ThermoFisher_TaqPathCOVID19_CE-IVD_KIT.pdf

9. Freire-Paspuel B, Vega-Mariño P, Velez A, Castillo P, Cruz M, Garcia-Bereguiain MA. Evaluation of nCoV-QS (MiCo BioMed) for RT-qPCR detection of SARS-CoV-2 from nasopharyngeal samples using CDC FDA EUA qPCR kit as a gold standard: An example of the need of validation studies. J Clin Virol. 2020;128: 104454.
